# Structural Insight into the MCM double hexamer activation by Dbf4-Cdc7 kinase

**DOI:** 10.1038/s41467-022-29070-5

**Published:** 2022-03-16

**Authors:** Jiaxuan Cheng, Ningning Li, Yunjing Huo, Shangyu Dang, Bik-Kwoon Tye, Ning Gao, Yuanliang Zhai

**Affiliations:** 1 State Key Laboratory of Membrane Biology, Peking-Tsinghua Joint Center for Life Sciences, School of Life Sciences, Peking University, Beijing, 100871 China; 2 School of Biological Sciences, The University of Hong Kong, Hong Kong, China; 3 Division of Life Science, The Hong Kong University of Science & Technology, Hong Kong, China; 4 Institute for Advanced Study, The Hong Kong University of Science & Technology, Hong Kong, China; 5 Department of Molecular Biology & Genetics, Cornell University, Ithaca, NY 14853 USA; 6 National Biomedical Imaging Center, Peking University, Beijing, 100871 China; 7 Present Address: Vaccine Research Center, National Institute of Allergy and Infectious Diseases, National Institutes of Health, Bethesda, MD USA

**Keywords:** Origin firing, Cryoelectron microscopy, Enzyme mechanisms

## Abstract

The Dbf4-dependent kinase Cdc7 (DDK) regulates DNA replication initiation by phosphorylation of the MCM double hexamer (MCM-DH) to promote helicase activation. Here, we determine a series of cryo electron microscopy (cryo-EM) structures of yeast DDK bound to the MCM-DH. These structures, occupied by one or two DDKs, differ primarily in the conformations of the kinase core. The interactions of DDK with the MCM-DH are mediated exclusively by subunit Dbf4 straddling across the hexamer interface on the three N-terminal domains (NTDs) of subunits Mcm2, Mcm6, and Mcm4. This arrangement brings Cdc7 close to its only essential substrate, the N-terminal serine/threonine-rich domain (NSD) of Mcm4. Dbf4 further displaces the NSD from its binding site on Mcm4-NTD, facilitating an immediate targeting of this motif by Cdc7. Moreover, the active center of Cdc7 is occupied by a unique Dbf4 inhibitory loop, which is disengaged when the kinase core assumes wobbling conformations. This study elucidates the versatility of Dbf4 in regulating the ordered multisite phosphorylation of the MCM-DH by Cdc7 kinase during helicase activation.

## Abstract (web summary)

The Dbf4-dependent kinase Cdc7 (DDK) is essential for eukaryotic DNA replication. Here, the authors present a series of cryo-EM structures elucidating the versatility of this kinase in exerting an ordered phosphorylation of its essential target to promote replication initiation.

## Introduction

In eukaryotes, DNA replication initiates from multiple origins along each chromosome^1,2^. Every initiation event is tightly regulated to ensure that it occurs only once per cell cycle. This regulation is achieved through a temporal separation of replication licensing from origin firing by the oscillating kinase activities during the cell division cycle^3,4^.

At early G1 phase, when the DDK (Dbf4-dependent Cdc7 kinase) and S-CDK (S-cyclin dependent kinase) kinase activities are low, the origin recognition complex (ORC) together with Cdc6 and Cdt1 load the Mcm2-7 helicases onto origin DNA to form an inactive MCM double hexamer (DH) encircling dsDNA to license replication^5–9^. Upon entering S phase, the rise of these kinase activities drives origin firing to initiate DNA replication^4^. During this process, DDK and S-CDK, act in concert with various origin-firing factors to convert the DH into two active Cdc45-Mcm2-7-GINS (CMG) helicases which then serve as scaffolds for the assembly of bidirectional replisomes^10–12^. Extensive genetic, biochemical, and structural studies have been carried out on the DDK and S-CDK, but little is known about their mechanistic actions on their substrates partly because the structures of these kinases in complex with the DH and other targets have not been determined.

Cdc7 is a serine/threonine kinase whose activity is dependent on its association with Dbf4^13^. The protein level of Dbf4 fluctuates during the cell cycle, peaking in late G1 and disappearing during mitotic exit to maintain a low DDK activity in G1 phase^14–16^. Although DDK can act on diverse substrates implicated in various events related to cell cycle progression^17–21^, its major target is the Mcm2-7 complex^22,23^. DDK prefers to phosphorylate Mcm2/4/6 subunits that have been incorporated into the DH at origin DNA^24,25^. Compelling evidence in budding yeast has also shown that the essential role of DDK in DNA replication is to relieve an inhibitory effect imposed on replication initiation from the N-terminal serine/threonine-rich domain (NSD) of Mcm4 (residues 74-174), as NSD deletion can bypass DDK function^26^. Furthermore, DDK function can also be bypassed by the P83L (bob1) mutation in Mcm5, which is not a substrate of DDK, suggesting that DDK phosphorylation may affect the overall structure of the MCM-DH^22^. However, previous structural studies at low resolutions did not detect an obvious conformational change in the core of the DH after DDK phosphorylation^7,27^. In response to DNA damage in S phase, Rad53 antagonizes DDK through inhibiting its kinase activity, keeping it away from unfired origins^28–30^.

Cdc7 orthologs share similar sequence signatures with many canonical serine/threonine kinases but with variable kinase insertions (KIs) intervening the conserved kinase elements^31^. In contrast, Dbf4 contains only three short regions, motifs N, M, and C, with limited homology among orthologs while the remaining parts are predicted to be largely unstructured. Nevertheless, the function of DDK in regulating replication initiation is highly conserved among eukaryotes.

Crystal structures of human Cdc7-Dbf4 kinase complex have been determined by removing most flexible motifs or insertions to optimize crystallization^32,33^. In these structures, Cdc7 assumes a bilobed configuration with its kinase active site sitting in a deep cleft in between the N- and C- lobes. Motifs M and C from Dbf4 each positions against one of the bilobed surfaces of Cdc7 to stabilize the kinase complex in an active state. These truncated Dbf4 structures, though informative, do not provide a complete picture about Cdc7 activation by Dbf4. It is also unclear how DDK is recruited onto the DH to target its substrates for phosphorylation and what impact DDK has on the conformation of the DH that signals helicase activation.

Cdc7’s role in regulating cell proliferation is most evident in cancer cells^34^. Cdc7 overexpression is often observed in human cancer cell lines and tumor tissues, and cancer cells with Cdc7 knockdown undergo apoptosis in a p53-independent manner^35^. Thus, Cdc7 has emerged as an attractive drug target for cancer therapy^36^. At present, a number of Cdc7 inhibitors are being developed or evaluated in clinical or preclinical trials. Notably, all of these inhibitors are ATP competitors with a potential to produce undesirable off-target effects through non-specific binding to other kinases. To achieve selective killing of only cancer cells for chemotherapy, it is important to develop highly specific DDK inhibitors that are capable of antagonizing unique features of the kinase. Therefore, understanding the detailed structural and molecular mechanisms of Cdc7 activation by Dbf4, the docking of the DDK onto the DH, and the activation of DH by DDK would be invaluable for developing anti-cancer drugs.

In this work, we assemble the full-length DDK onto the endogenous MCM-DH purified from G1 chromatin of budding yeast and determine the cryo-EM structures of the kinase-substrate complexes. The structures present snapshots of the Dbf4-Cdc7 kinase engaging with the DH in an intricate process that regulates DDK substrate targeting and helicase activation at origin DNA.

## Results

### Structural determination of DH-DDK complexes

The DDK has been shown to specifically target MCM subunits within the assembled DH^24^. To investigate how DDK performs this function, we isolated the full-length Cdc7-Dbf4 heterodimer from yeast cells, and assembled the kinase complex onto the DH in the presence of ATP or ATP-γ-S for structural analysis. Initially, we incubated DDK in excess with the DH at a molar ratio of 10:1 before subjecting the mixture to cryo sample preparation to capture the kinase-substrate complex in different functional states. However, the population of the DDK-bound DH (referred to as DH-DDK) was less than 10% of the total particles for both the ATP and ATP-γ-S treated samples (Supplementary Fig. 1a–c). Similar results were observed when the kinase complex was incubated with two mutant DHs, DH-Mcm4ΔN140 (referred to as DHΔN140) and DH-Mcm4ΔN174 (referred to as DHΔN174) (Supplementary Fig. 1d, e), in which the NSD was either partially or completely truncated from Mcm4. These results indicate that the interaction between DDK and the DH is relatively weak and transient.

To stabilize the assembled DH-DDK complexes, we subjected the kinase-substrate complex (treated with ATP-γ-S) to crosslinking by grafix, which involved a mild fixation with glutaraldehyde (GA) during ultracentrifugation^37^. Using the samples collected from grafix for cryo-EM analysis, we found that the percentage of DDK-bound DH particles was greatly increased to 88.7% of the total particles (Supplementary Fig. 3a–e), indicating that the GA crosslinking effectively preserved the integrity of the unstable kinase-substrate complexes. As evident from 2D classification averaged images, many clearly show densities for bound DDK (Supplementary Fig. 2c). Subsequent initial 3D reconstruction showed that while the DH is relatively rigid, DDK exhibits high flexibility (Supplementary Fig. 2d). Considering that the two binding sites of DDK on a single DH is symetrical, we used symmetry expansion to double the particle number and performed focused classification on the DDK region (Supplementary Fig. 2d). The first round of 3D classification identified two major conformational subsets for DDK on the DH. Based on the occupancy of the two symmetrical DDK binding sites on the DH and the general stability of DDK (stable DDK vs stable Dbf4-NTD) (Supplementary Fig. 2d), the particles of these two subsets could be divided into six groups (Supplementary Fig. 3), representing major compositional and conformational populations of DH-DDK assemblies in the reaction. Consequently, the maps of these six groups were determined at resolutions ranging from 3.4 to 4.0 Å (Supplementary Fig. 3g). Groups I-III, representing DHs with two DDKs bound, account for 44.1% of the total particles (Supplementary Fig. 3a–c). In these groups, the two DDKs are well separated from each other on the DH, showing no detectable interaction between the kinase complexes. Groups IV and V, showing one DDK bound to the DH, account for 44.5% of the total particles (Supplementary Fig. 3d, e); whereas Group VI (11.3%) represents DHs without DDK bound (Supplementary Fig. 3f). Together, these DH-DDK structures present snapshots of the docking of two DDKs onto the DH to activate the MCM complexes.

**Fig. 1:**
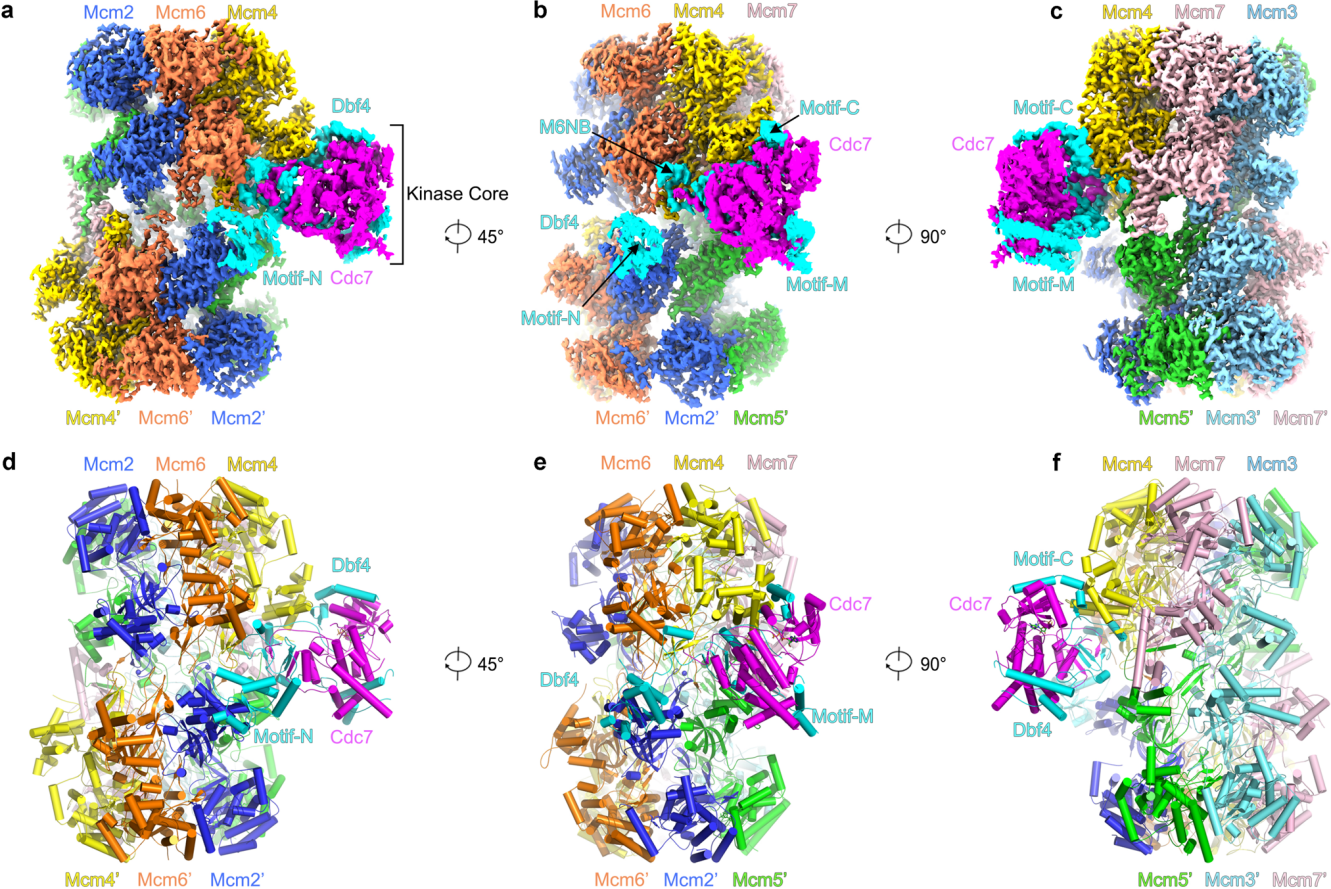
Overall structure of MCM double hexamer bound with DDK. **a**–**c** Side views of the segmented cryo-EM density map of the DDK-DH (State I) displayed with indicated rotations along the cylinder axis. **d**–**f** Same as (**a**–**c**) respectively but shown with the cylindrical atomic model. MCM subunits, Dbf4, and Cdc7 subunits are color-coded and labeled as indicated. The positions of the motifs N, M, and C of Dbf4 and the kinase core of DDK are also labeled and highlighted.

In the map produced from particles of the first subset, both Dbf4 and Cdc7 are stably attached to the DH (State I) (Fig. 1, Supplementary Fig. 2d, and Supplementary Movie 1), whereas in the second subset only the N-terminal half of Dbf4 is visible in the derived maps. Particles of the second subset were then pooled and subjected to another round of focused 3D classification, which identified several additional states for DDK (States II-IV). In the maps of States II-IV, the N-terminal part of Dbf4 are stably bound at the same site, while the kinase core of Cdc7 is in different wobbling positions (Supplementary Fig. 2d).

The final density map of State I has a global resolution of 2.9 Å (Supplementary Fig. 2d–f) with most residues at the DH-DDK interfaces clearly resolved (Supplementary Fig. 2g). However, the local resolution of the kinase core region, which is away from the DH-DDK interface, was still relatively low. Based on signal subtraction focusing on the DDK region, we further improved the map of the kinase core region of DDK to a resolution of 3.8 Å (Supplementary Fig. 2d–f), enabling atomic modeling for ~50% of the sequence of Dbf4 and ~90% of Cdc7.

### The role of Dbf4 in docking Cdc7 onto the DH

**Fig. 2:**
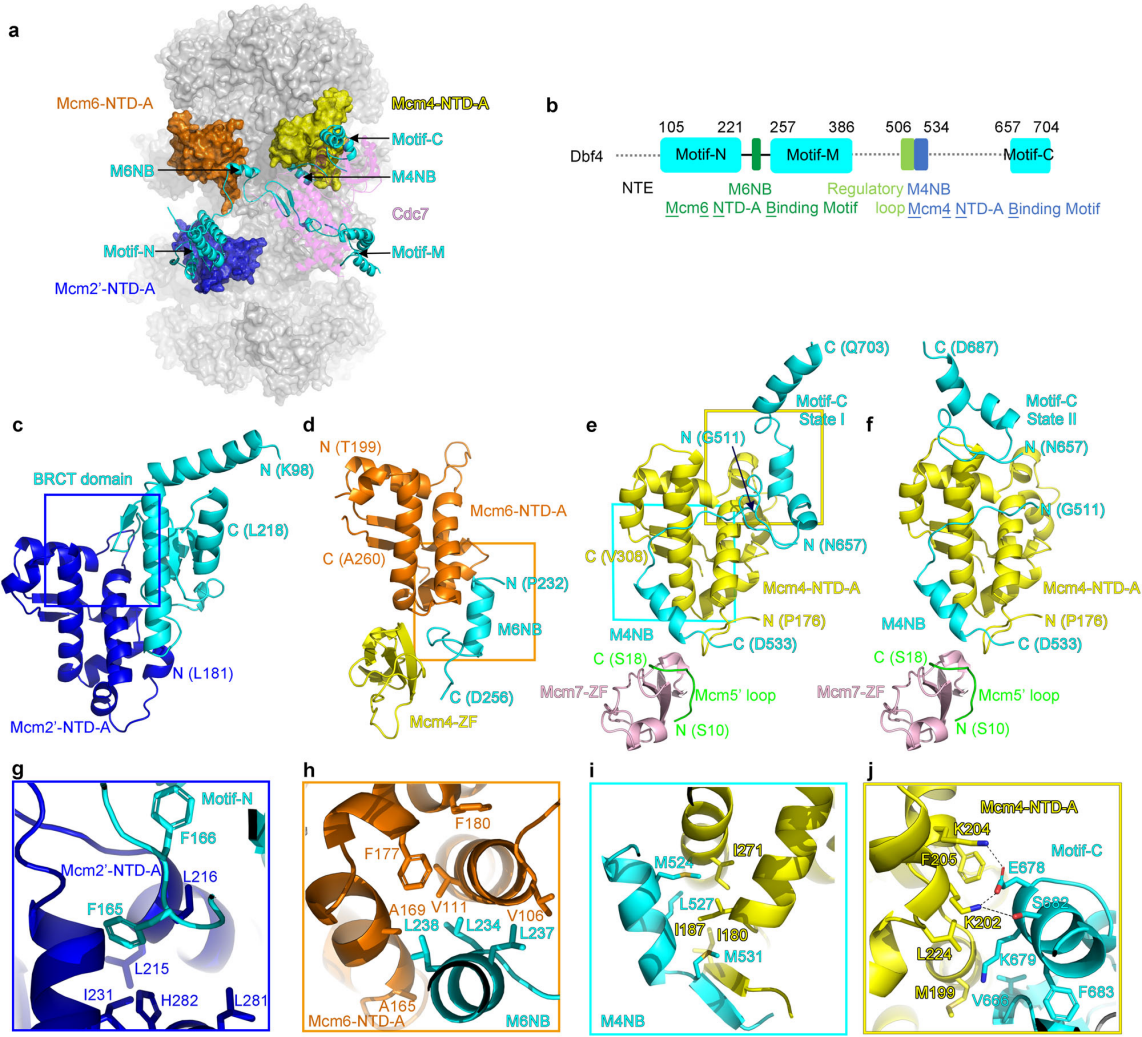
Distinct segments of Dbf4 mediate the docking of DDK onto the DH. **a** Side view of the DH-DDK structure with MCM subunits shown in surface presentation and DDK in cartoon presentation. For clarity of Dbf4 segments, Cdc7 is displayed in transparency. The NTD-As of Mcm2, Mcm6, and Mcm4, Dbf4, and Cdc7 are color-coded and labeled. **b** Schematic domain organization of Dbf4. Dashed lines denote highly disordered segments which are not resolved in the DH-DDK structure. **c** Dbf4 motif N binds to the NTD-A of Mcm2 using its BRCT domain. The NTE (residues 1-180) of Mcm2 is highly flexible in this structure. The resolved N- and C-terminal residues are labeled as indicated. **d** Dbf4-M6NB motif is docked onto the NTD-A of Mcm6. **e**, **f** The NTD-A of Mcm4 interacts with the M4NB and motif C of Dbf4. The ZF of Mcm7 and the very N-terminal loop of Mcm5’ also show weak interaction with the M4NB motif. Two distinct binding surfaces on Mcm4-NTD-A can be found for Dbf4 motif C from States I (**e**) and II (**f**) of the DH-DDK structure. **g**–**j** Magnified views of the boxed regions in (**c**–**e**), highlighting the interaction details of Dbf4 with the NTD-As. Side chains of selected residues at the interfaces are shown in stick model.

A striking feature of the DH-DDK structure is that Cdc7 has almost no physical contact with the DH (Fig. 1). Instead, the association of DDK with the DH is mediated exclusively by Dbf4 through multiple contacts with the NTD-A subdomains (NTD-As) of three MCM subunits, Mcm4, Mcm6 and Mcm2’ from the opposite hexamer (Figs. 1 and 2a, Supplementary Movie 1). This configuration explains the substrate preference of DDK for the DH, as both MCM hexamers contribute to the recruitment of each Dbf4.

**Fig. 3:**
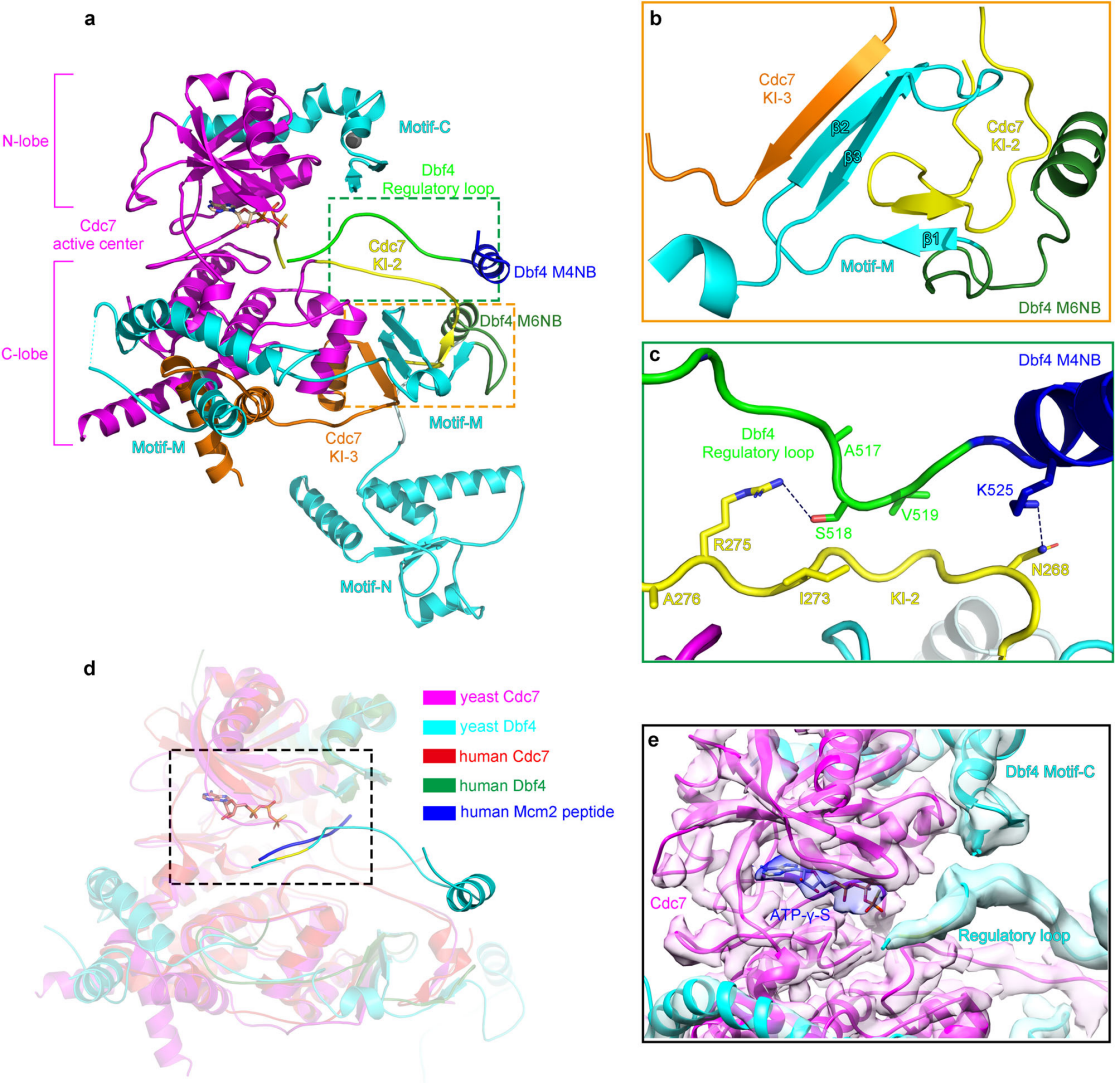
Structural features of the kinase core of DDK from the assembled DH-DDK. **a** Overview of the Dbf4-Cdc7 structure shown in a cartoon view with the motifs N, M, and C of Dbf4 in cyan, the M6NB motif of Dbf4 in forest green, the M4NB motif of Dbf4 in blue, the regulatory loop of Dbf4 in green, the canonical kinase region of Cdc7 in magenta, the kinase insertion 2 (KI-2) in yellow and 3 (KI-3) in orange. Zn atom is shown as grey sphere and nucleotide (ATP-γ-S) in stick model. **b** Magnified view of the boxed region in **a** showing three β-strands (β1-3) from Dbf4 motif M engaging with KI-2 and KI-3 of Cdc7 to maintain these kinase insertions in stretched conformations. **c** Magnified view of the boxed region in (**a**) highlighting the interactions between KI-2 of Cdc7 and the regulatory loop and M4NB of Dbf4. The hydrogen bonds between R275 of Cdc7 and S518 of Dbf4, N268 of Cdc7 and K525 of Dbf4 are indicated by dashed lines. **d** Superimposition of the yeast Dbf-Cdc7 with a truncated version of human CDC7-DBF4 in complex with an Mcm2 (residues 38–46) peptide substrate (PDB code 6YA7)^33^. The structures are shown in cartoon representation and color-coded as indicated. The postions of Thr506 and Ser507 residues from Dbf4 are highlighted in yellow. **e** Magnified view of the boxed region in (**d**) but with the cryo-EM map superimposed. The γ-phosphate of the ATP-γ-S nucleotide (blue) is situated in the active center of Cdc7 and very close to the last resolved N-terminal residue of Dbf4 regulatory loop (cyan).

Dbf4 is a very flexible protein (704 residues) containing three long disordered segments, and only those segments interacting with MCM subunits or Cdc7 are resolved in our structure (Fig. 2a, b). These binding motifs spread over the entire length of the protein (Fig. 2b). At the N-terminus, motif N of Dbf4 (residues 105–221) containing a BRCT domain clasps onto the NTD-A of Mcm2’ (Figs. 1a, d and 2a). Consistent with the crystal structure of the human DDK complex^33^, motif M exclusively interacts with Cdc7 (Figs. 1b, c, e, f, and 3a). In contrast, motif C of Dbf4 at its very C-terminus is sandwiched between Cdc7 and the NTD-A of Mcm4 (Fig. 1c, f), appearing to stabilize DDK on the DH. Of note, motif C adopts different orientations relative to Mcm4-NTD-A in States I and II of the DH-DDK (Fig. 2e, f). In addition to these known motifs, two other MCM-interacting motifs of Dbf4 were also identified in the DH-DDK structure. One is a short α-helix containing sequence (residues 230–257) (referred to as Mcm6-NTD-A binding motif (M6NB)), which connects motifs N to M across the hexamer junction, and docks onto the subunit interface between the NTD-A of Mcm6 and the zinc finger motif (ZF) of Mcm4 (Figs. 1b, e and 2b, d). The other is also a short helix (residues 522-534) situated on the NTD-A of Mcm4 (referred to as Mcm4-NTD-A binding motif (M4NB)) (Fig. 2b, e, f), interacting with the first β-strand and nearby α-helices of Mcm4-NTD-A, the ZF of Mcm7 and the very N-terminal loop of Mcm5’ (Fig. 2e, f). Interestingly, these Dbf4 motifs recognize distinct surfaces of similar NTD-A subdomains from different MCM subunits (Fig. 2c–f). While the interfaces of MCM subunits with motifs N, M6NB, and M4NB of Dbf4 are largely hydrophobic (Fig. 2g–i), the interactions between motif C and Mcm4-NTD-A are contributed by both hydrophobic and hydrophilic interactions (Fig. 2j). Motifs N, M, and C^31,38,39^ are conserved across species, suggesting that yeast and higher eukaryotes share a similar strategy for DDK docking to anchor the kinase complex onto the DH and place the catalytic Cdc7 subunit proximal to the NTD-A of Mcm4, where its only essential substrate, Mcm4-NSD, is located (Figs. 1 and 2a).

When motif N is removed from Dbf4, the ability of DDK to phosphorylate MCM complex is largely suppressed (Supplementary Fig. 4a–c). In contrast, removing the M6NB of Dbf4 allows Cdc7 to retain a kinase activity comparable to the WT DDK (Supplementary Fig. 4b, d). These results indicate that the interaction between motif N of Dbf4 and Mcm2-NTD-A is crucial for DDK recruitment onto the DH to function. A recent report suggests that the NTE of Mcm2 also plays an important role in DDK recruitment^30^. However, the Mcm2-NTE is highly flexible in our structure. It is likely that this floppy element of Mcm2 contributes to the initial docking of Dbf4 onto the NTD-A of Mcm2 through a transient interaction with Dbf4 motif N or other region(s) from DDK.

### The structure of DDK on the DH

Overall, the structure of the kinase core of yeast DDK matches very well with its human counterpart (Fig. 3d) despite their limited Dbf4 homology^31^. Their structural similarity indicates that DDK employs a highly conserved mechanism to target its substates on DHs across species. In the DH-DDK structure, the yeast Cdc7 adopts a bilobed configuration with the active center located in a deep cleft between its N- and C-lobes (Fig. 3a), a common feature shared among most serine/threonine kinases^40^. Similar to the human DDK structure^32^, the N- and C-lobes of Cdc7 are positioned against motif M and motif C of Dbf4, respectively (Fig. 3a), suggesting that Dbf4 may have a role in modulating the conformation of Cdc7 kinase. Indeed, motif M plays an important role in stabilizing two large kinase-insertion loops (KI-2 and KI-3) on the Cdc7 C-lobe (Fig. 3a, b). Specifically, as also seen in the human DDK structure^33^, a β-hairpin (β2-3) of Dbf4 motif M pairs up with the N-terminal end of the KI-3 of Cdc7; and another short β-strand (β1) of the motif M complements a short β-strand from the KI-2 in an anti-parallel fashion (Fig. 3b). This special arrangement stabilizes the KI-2 and KI-3 in a stretched conformation, contributing to a structured activation loop of the kinase in a configuration resembling that observed in the active human DDK^33^. In addition, two newly identified α helices, which extend from the C-terminus of motif M, encircle the C lobe of Cdc7 for more than a half turn to associate with most of the α helices present in this domain (Fig. 3a), establishing a strong coupling between Dbf4 and Cdc7. Together, the extensive interactions of Dbf4 with the two lobes of Cdc7 as well as its special engagement with the KI-2 and KI-3 stabilize, shape as well as activate the Cdc7 kinase.

More importantly, in our DH-DDK (State I) structure, a unique loop from Dbf4 is found residing in the substrate-binding pocket of Cdc7 (Fig. 3a), in an equivalent position of the peptide substrate in the human DDK structure^33^ (Fig. 3d). This loop (residues 504–519) extends from the N-terminus of the M4NB motif, and interacts with the KI-2 in several positions (Fig. 3c). The N-terminal residue of this loop, Thr506, is situated close to the γ-phosphate of the ATP-γ-S nucleotide in the active center of Cdc7 (Fig. 3d, e). This observation suggests that this loop (refered as regulatory loop) likely has a regulatory role to limit the accessibility of the stubstrate binding pocket.

### Dynamic association of the kinase core with the NTD-A of Mcm4

**Fig. 4:**
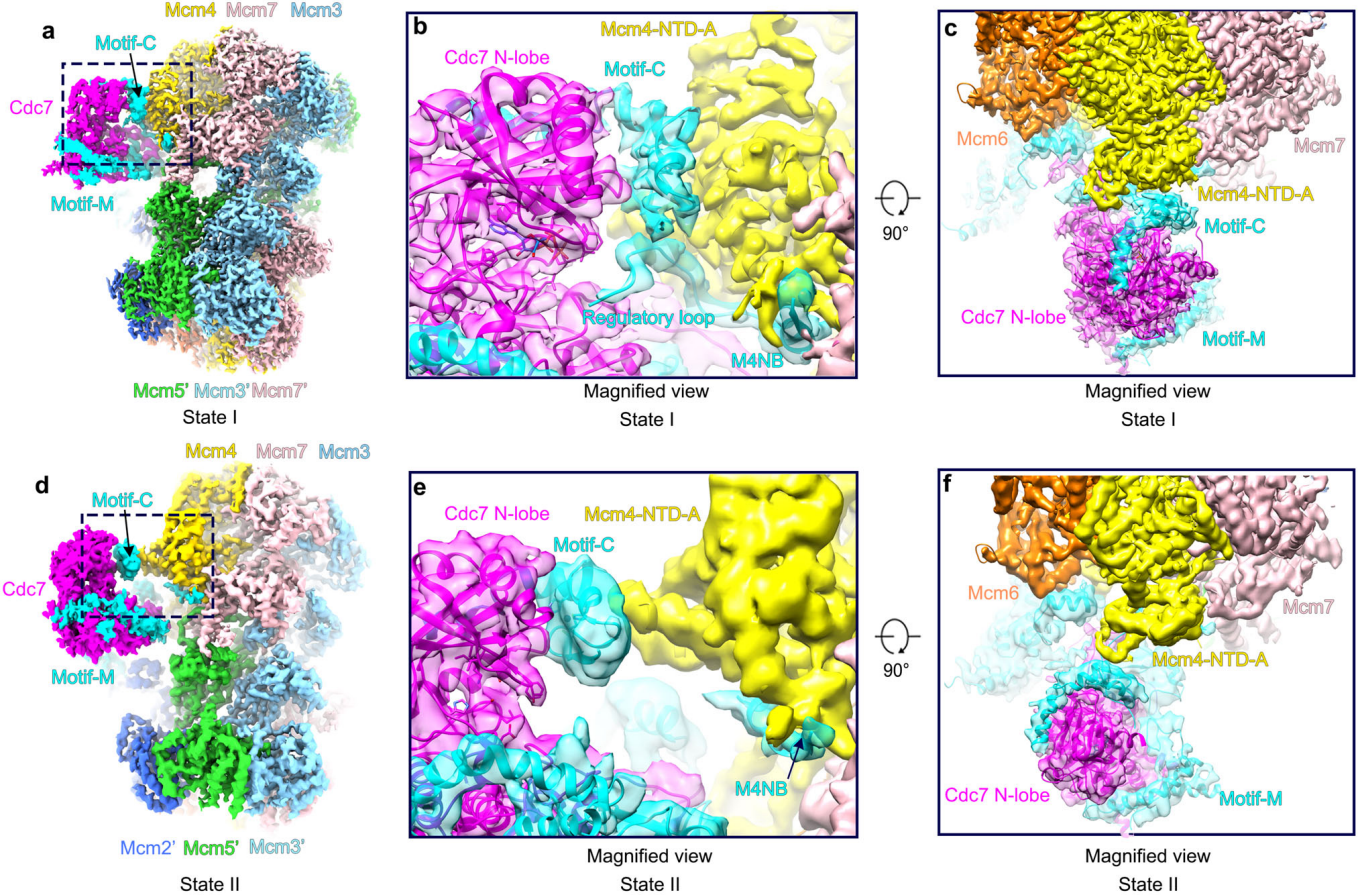
Conformational change of kinase core relative to the NTD-A of Mcm4. **a** Side view of the cryo-EM density map of the DH-DDK complex (State I). **b** Magnified view of the boxed region in (**a**), highlighting the kinase core of DDK bound to the NTD-A of Mcm4 via motif C of Dbf4. **c** Same as (**a**) but with a 90° rotation and shown in top view with a focus on Mcm4 and DDK. **d**–**f** Same as (**a**–**c**) but shown in State II of the DH-DDK structure.

Motif C of Dbf4 mediates the attachment of the kinase core to the NTD-A of Mcm4 (Figs. 1c, f and 4) so that the active center of Cdc7 is oriented towards Mcm4 for the capture of Mcm4-NSD with a higher probability than the other nearby substrates (Figs. 1 and 4). When motif C of Dbf4 is removed, the activity of the mutant DDK is significantly compromised such that phosphorylation of both Mcm4 and Mcm6 is suppressed (Supplementary Fig. 4g). These results suggest that motif C of Dbf4 plays a critical role in enabling the kinase activity.

More interestingly, while the motifs N and M6NB of Dbf4 remain stably bound to the analogous surfaces on the NTD-As of Mcm2’ and Mcm6, the kinase core of DDK takes up several distinct positions, in two relatively stable states (I and II) (Fig. 4), two less stable states (III-IV) and more highly dynamic states in different subpopulations of the DH-DDK particles (Supplementary Figs. 2d, 5). In State I, the kinase core makes an intimate contact with the Mcm4-NTD-A via motif C of Dbf4 (Fig. 4a–c). In State II, the kinase core still engages with the NTD-A of Mcm4 but undergoes a rotation, which relocates the ZF motif from motif C of Dbf4 to a different surface on Mcm4 (Fig. 4d–f, Supplementary Movie 2). In this configuration, the kinase core is weakly attached to Mcm4 as if separated from its docking site. In contrast, in States III–IV, the kinase core appears relatively unstable while Dbf4 shows strong interactions with Mcm2’ and Mcm6 (Supplementary Fig. 5). Notably, the kinase core from these two less stable states is completely disengaged from Mcm4 such that the main body of Cdc7 is turned away from Mcm4, and positioned towards the NTD-A of Mcm2 where the motif N of Dbf4 binds (Supplementary Fig. 5c, d, g, h, k, l). In State IV, the EM density of the kinase core makes a direct contact with motif N of Dbf4 (Supplementary Fig. 5d, h). These different conformations of DDK illustrate a dynamic association between the kinase core and the NTD-A of Mcm4, and explain the ability of DDK to target various sites within the NTEs of Mcm2, Mcm4, and Mcm6 on the DH.

In State I of the DH-DDK, the regulatory loop (residues 504–519) of Dbf4, which is upstream of the M4NB (Fig. 3a), is embedded in the substrate binding site of Cdc7 (Fig. 3a, d, e). Similar confirmation of this loop can also be observed in both DHΔN140-DDK and DHΔN174-DDK structures (Supplementary Fig. 6). This feature suggests a regulatory function for this loop on the activity of Cdc7 kinase. Intriguingly, this putative inhibitory loop does not associate with the kinase in State II of the DH-DDK, whereas its downstream M4NB remains stably attached to the NTD-A of Mcm4 (Fig. 3d, e). These comparisons suggest that the M4NB serves as a relatively fixed anchor point for attaching DDK to the NTD-A of Mcm4, whereas the regulatory loop is a dynamic element that might impact the substrate preference of Cdc7. Indeed, upon removal of the regulatory loop, the mutant DDK appears to be more active towards Mcm6 and the phosphorylation of Mcm4 is also evidently delayed (Supplementary Fig. 4e)

### DDK dislodges Mcm4-NSD on the DH

**Fig. 5:**
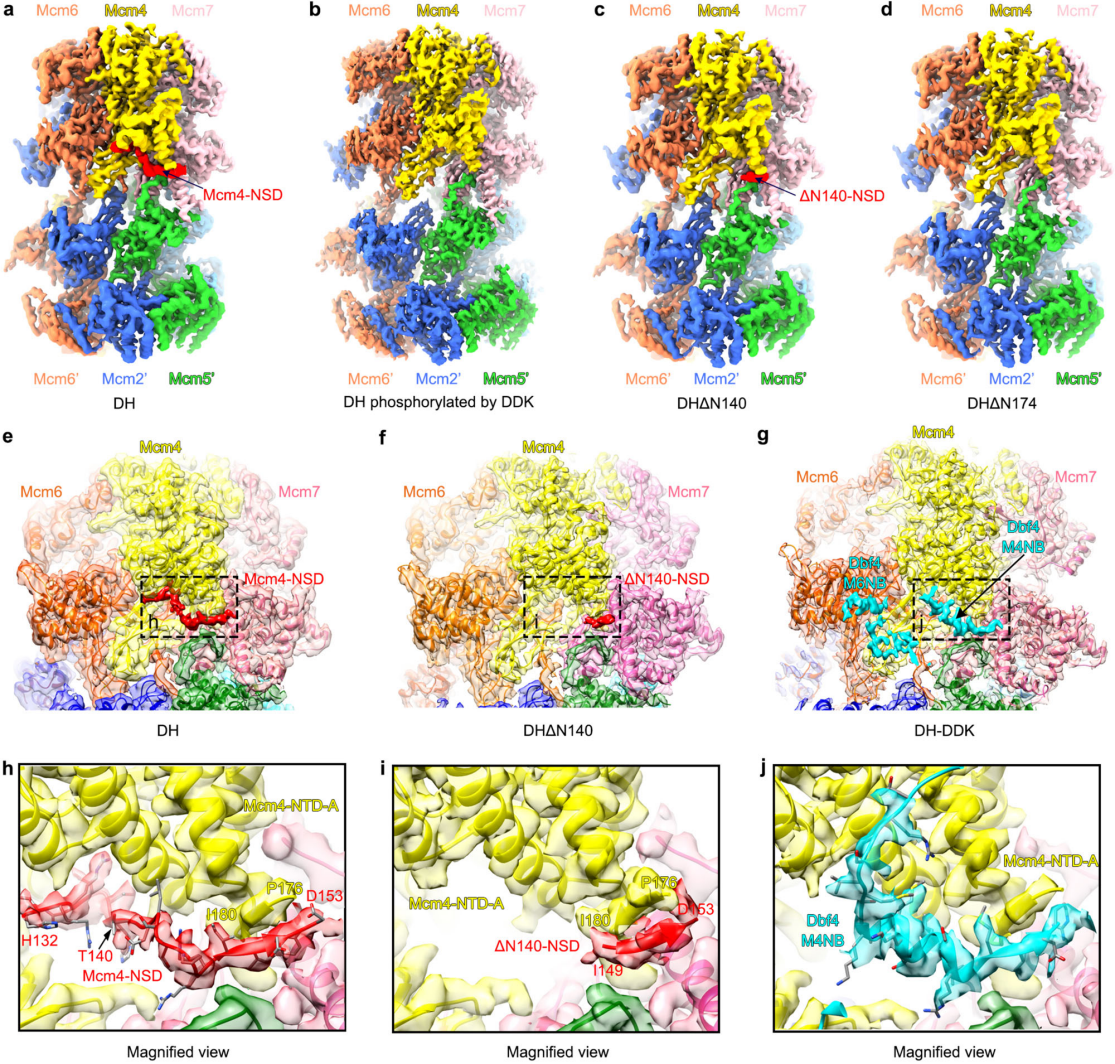
Dbf4 displaces the NSD of Mcm4 on the DH. Side views of the cryo-EM density maps of the WT DH before (**a**) and after (**b**) DDK phosphorylation and the two mutant DHs, DHΔN140 (**c**) and DH-ΔN174 (**d**). The structured fragments of Mcm4-NSD are highlighted in red. **e, f** Zoomed-in views of (**a**) and (**c**) with the atomic model of the DH superimposed, highlighting the structured NSD on the NTD-A of Mcm4. **g** Zoomed-in view of the cryo-EM map of the DH-DDK (State I) with the atomic model superimposed. The kinase core is removed to highlight the binding of the M4NB and M6NB motifs of Dbf4 to the NTD-As of Mcm4 and Mcm6. Note that the M4NB of Dbf4 and the NSD of Mcm4 share an overlapping binding surface on the NTD-A of Mcm4. **h**–**j** Magnified views of the boxed regions in (**e**–**g**) respectively.

Previous study showed that the essential role of DDK is to relieve the inhibitory effect of Mcm4-NSD upon origin firing^26^. To understand the role of DDK phosphorylation in this process, we analyzed the structures of the NSD on the DH surface before and after DDK binding. In two published cryo-EM maps of the yeast DH, some extra densities associating with the NTD-A of Mcm4 were visible but not analyzed because of their poor quality for subunit identification and sequence assignment^7,9^. In our structures of the free DH (more than 90% of all particles) from the two large datasets of non-crosslinked DH-DDK treated with ATP-γ-S, similar EM densities were also observed. After multiple rounds of local 3D classification of this region (Supplementary Fig. 1a, b), we finally obtained a density map for the DH with an obviously structured fragment nested on the NTD-A surface of Mcm4. The map was resolved at an overall resolution of 3.2 Å (Supplementary Fig. 1a, b, f), enabling us to unambiguously assign residues 132–153 of the Mcm4-NSD to this density piece (Fig. 5a, e, h). The NSD fragment, largely in a loop conformation, wraps around the NTD-A of Mcm4 and extends to the subunit interface between Mcm4 and Mcm6. Its N-terminal residues before residue 131 appear highly disordered (Fig. 5a, e, h). The N-terminus of the structured NSD (residues 132-141) forms a U-turn loop at the juncture of the OB and NTD-A subdomains of Mcm4. The C-terminus of this fragment (residues 145-153) contains a short β-strand which is parallel to the N-termimal β-strand of the Mcm4-NTD-A (residues 176-181), leaving the connecting residues of 154-176 in a highly flexible loop.

To further confirm this assignment of the Mcm4-NSD, we next examined the structures of the two mutant DHs (DHΔN140 and DHΔN174) (Supplementary Fig. 1d, e). As shown in Fig. 5c, the EM densities for the residual NSD region (residues 148–153) in the map of the DH-ΔN140 is also evident (Fig. 5c, f, i). In contrast, when the entire NSD is truncated from Mcm4, the densities corresponding to the structured NSD completely disappear from the map of the DH-ΔN174 (Fig. 5d).

In the previous cryo-EM map of DH after DDK phosphorylation (EMD-3833)^7^, the entire NSD motif becomes unstructured and is no longer visible on the DH altogether. Consistently, in our dataset of non-crosslinked DH-DDK treated with ATP, where DDK phosphorylation is active, we did not identify any 3D classes with a structured Mcm4-NSD (Fig. 5b; Supplementary Fig. 1c). As removal of the NSD from Mcm4 bypasses the requirement of DDK for helicase activation^26^, we believe that Mcm4-NSD either deleted or phosphorylated by DDK achieves the same goal of remodeling NSD conformation to alleviate its inhibitory effect on helicase activation.

In addition, the dynamic behavior of Mcm4-NSD is best illustrated by comparing the DH structure before and after DDK binding. In the DH-DDK structure from the ATP-γ-S treated sample, upon DDK binding, Mcm4-NSD becomes flexible and completely invisible. Instead, the M4NB of Dbf4 (residues 520-534) associates with the Mcm4-NTD-A by displacing Mcm4-NSD from its binding site in the free DH (Fig. 5e–j), suggesting that the M4NB and Mcm4-NSD compete for the same binding surface on Mcm4. The phosphorylation of NSD likely acts to maintain this dislodged conformation after DDK is released from the DH.

### Temporal order of DDK phosphorylation of the MCM subunits

The structural features of the DH-DDK complex suggest that Dbf4 helps to establish a hierarchy for Cdc7 kinase to phosphorylate the multiple substrates located on the MCM complex. To test this hypothesis, we first examined the phosphorylation status of the MCM subunits by DDK. As DDK is very active in phosphorylating MCM subunits^27^, kinase assay was performed with purified DDK and DH on ice to slow down the reaction. Under this condition, a clear separation of the temporal phosphorylation patterns between Mcm4 and Mcm6 can be observed (Supplementary Fig. 4b). Phosphorylation of Mcm4 by DDK started immediately after the kinase was mixed with the DH in the presence of ATP. In sharp contrast, obvious phosphorylation of Mcm6 could only be detected at much later time points, and even up to 32 min a large portion of Mcm6 remained unphosphorylated (Supplementary Fig. 4b). Interestingly, when either the regulatory loop or M4NB of Dbf4 is removed, this temporal order of DDK in targeting its substrates is largely abolished, and phosphorylation of Mcm6 is much enhanced (Supplementary Fig. 4e, f). Together, our structural and biochemical data indicate that Dbf4 mediates the docking of DDK onto the DH to initiate an orchestrated series of phosphorylation events that begins with the NSD of Mcm4 containing the only essential phosphorylation targets for DDK in helicase activation.

## Discussion

The head-to-head dimeric MCM helicases encircling dsDNA are activated simultaneously to enable bidirectional DNA synthesis by passing each other along opposite single DNA strands to form the bidirectional replication forks. A previous working model proposed that the dimerization of two DDK complexes upon binding to the DH coordinates the simultaneous phosphorylation of the coupled MCM complexes^30^. However, in our DH-DDK structures, the DDKs are positioned apart from each other without direct contacts (Supplementary Fig. 3a–c). We did not capture any interactions formed between the assembled kinases or allosteric changes induced by one DDK to recruit the other, suggesting that individual DDKs might dock onto the DH in a stochastic manner to operate independently. However, we cannot rule out the possibility that two DDKs are loaded cooperatively through weak and/or transient interactions between them or through mediation of other factors. Indeed, forkhead transcription factor Fkh1 is known to recruit Dbf4 to a subset of early replication origins for helicase activation^41^. This example highlights the importance of specific DNA element(s), such as Fkh1 binding sites, in facilitating DDK docking onto the DH especially in cells where Dbf4 level is limited. It is also possible that phosphorylation of the DH by the first DDK might pave the way for recruiting the second DDK. These possibilities need to be further investigated by other approaches. Consistent with previous data that the DH structures with or without DNA bound appeared almost identical^7–9^, our structures, at much-improved resolution, confirmed that DDK binding and phoporylation did not induce obvious structural changes to the DH. It is likely that at least under in vitro condition, dsDNA bound with the DH does not play a major role in regulating DDK recruitment or its activity on the DH. An immediate consequence of DDK phosphorylation is the recruitment of Sld3-Sld7 and Cdc45 onto the DH^11,42^. Previous studies showed that the Sld3 and Sld7 heterodimers form a quaternary complex through dimerization of the CTD of Sld7^43^. It is likely that simultaneous activation of the two MCM hexamers is initiated by the recruitment of the Sld3-Sld7 dimer to the fully DDK-phosphorylated DH and not before.

DDK prefers to phosphorylate the NTEs of Mcm2, Mcm4, and Mcm6 in the context of the DH with the NSD of Mcm4 as the only essential substrate^24,26^. The docking of Cdc7 onto the DH is mediated exclusively by Dbf4 through engaging with the NTD-As of Mcm2 from one hexamer and Mcm4 and Mcm6 from the opposite hexamer (Figs. 1 and 2). This arrangement ensures DDK to preferentially target MCM subunits assembled into the DH, and also positions the kinase core in a strategic location to capture the NSD of Mcm4 with a higher probability (Figs. 1 and 4, Supplementary Fig. 4b). Specifically, the M4NB motif of Dbf4 displaces the NSD from its binding surface on Mcm4 through competitive binding to facilitate an immediate capture of NSD by the kinase. Importantly, the kinase core of DDK can assume multiple wobble conformations (States II-IV) when disengaging from Mcm4. In fact, a large population of the DH-DDK particles exhibit a highly dynamic kinase core that cannot be modeled while motif N and M6NB of Dbf4 remain stably associated with Mcm2 and Mcm6. This dynamic feature enables the kinase to reach out for its substrates from Mcm2 and 6. After DDK phosphorylation, the NSD motif becomes highly flexible and loses its affinity for Mcm4-NTD-A. This is highly consistent with the observation that NSD deletion can bypass the requirement of DDK for helicase activation^26^. Our observation also agrees with a previous study which showed that a phosphomimetic mutant of Mcm4 with seven DDK-dependent phosphorylation sites mutated to D or E, can support growth of a *cdc7-4* strain at nonpermissive temperature^44^. In fact, two of these DDK sites, T140 and S141, are located right in the middle of the structured Mcm4-NSD (residues 132–153) (Fig. 5h). It is likely that DDK phosphorylation of these two sites are important for preventing the NSD from binding to the NTD-A of Mcm4 and facilitates its displacement by Dbf4-M4NB. These results suggest that the inhibitory effect of the NSD on helicase activation may modulate the accessibility of particular surfaces on Mcm4 to helicase-activating factors before S phase entry. In further support of this notion, Tof1, a component of fork protection complex (FPC), binds to these same surfaces on Mcm4 in the replisome in addition to docking on the NTD-As of Mcm2 and Mcm6 (Supplementary Fig. 7a–d)^45^. It is also important to note that Sld3 binds to the phosphorylated NTEs of Mcm4 and Mcm6 to promote the recruitment of Cdc45 onto the DH for helicase activation upon DDK phosphorylation^42^. Further investigation is needed to determine how Sld3-Sld7 complex and/or other helicase-activating factors such as Cdc45 are recruited onto the DH through the NSD protected surfaces, as well as the potential roles of these phosphopeptides from Mcm4 and Mcm6.

A recent in vitro study showed that Mcm2-NTE (1-127) plays a critical role in recruiting DDK onto the DH as removal of this motif from Mcm2 largely disrupted this process^30^. In addition, the NTE of Mcm2 is also found to be important for mediating the interaction between Mcm2 and Dbf4 in a yeast two-hybrid analysis^46^. However, the NTE of Mcm2 is highly flexible and not visible in our DH-DDK structures. How Mcm2-NTE functions in DDK recruitment remains obscure. Notably, this floppy NTE is connected to the NTD-A of Mcm2 which serves as a binding platform for the BRCT domain of Dbf4-motif N. Removal of the BRCT domain from Dbf4 significantly reduces the kinase activity of DDK towards Mcm4 (Supplementary Fig. 4c), indicating that the binding of Dbf4 motif N to the NTD-A of Mcm2 is critical for docking the DDK. We speculate that Mcm2-NTE may guide the BRCT domain of Dbf4 onto the NTD-A of Mcm2 through a weak and transient interaction. BRCT domains are known to bind phosphopeptides^47^. Previous studies have reported that priming phosphorylations of the MCM complex by other kinases are required for DDK recruitment, and eliminating those primed phosphorylation by phosphatase treatment largely suppresses DDK from binding to the DH^24,44^. Indeed, multiple primed phosphorylation sites have been identified in the NTE of Mcm2^44^. Mcm2-NTE primed with phosphorylations might serve as bait for trapping the BRCT domain of Dbf4 before docking onto the NTD-A of Mcm2. Alternatively, the NTE of Mcm2 might be required to modulate the NTD-A of Mcm2 in a state competent for the binding of Dbf4-motif N.

**Fig. 6:**
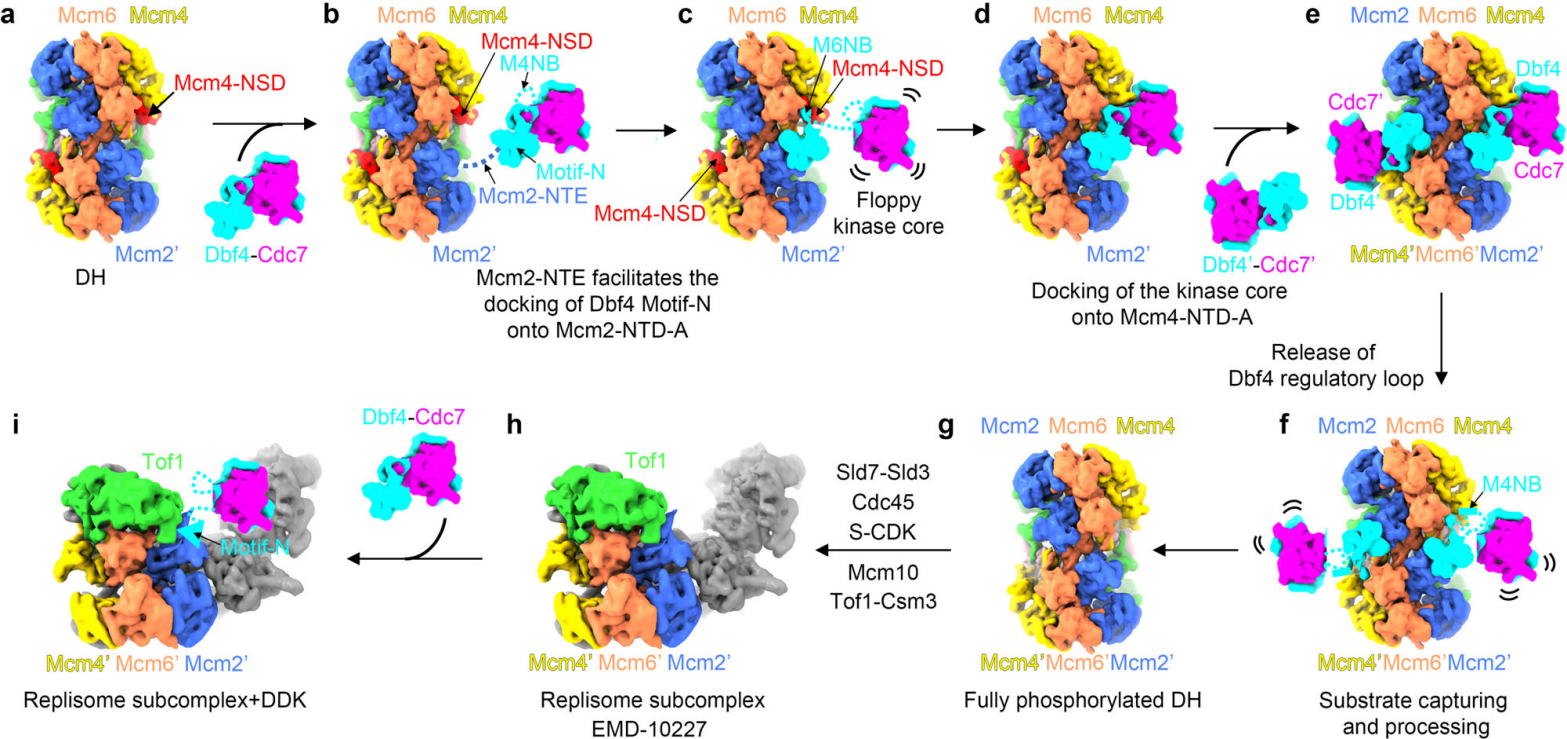
Model illustrating the action of DDK on Mcm2-7 complex to regulate helicase activation and replisome activities. To summarize the high-resolution snapshots of the DH-DDK structures into a congruent series of motion frames that represent the step-by-step action of DDK on its MCM substrates. The low-pass filtered cryo-EM maps are used to depict DH, DDK, and replisome (EMD-10227). **a** Once assembled at replication origin, the DH encircles dsDNA with the NSD of Mcm4 nested on its own NTD-A to impose an inhibitory effect on helicase activation. **b** The unstructured NTE of Mcm2’ engages with motif N of Dbf4 and/or other region of DDK to facilitate the docking of motif N onto the NTD-A of Mcm2’. **c** When motif N and M6NB of Dbf4 bind to the NTD-As of Mcm2’ and Mcm6 respectively, the floppy kinase core of DDK is positioned proximal to the NTD of Mcm4. **d** The kinase core is then docked onto the NTD-A of Mcm4 with the NSD of Mcm4 displaced by the M4NB of Dbf4 to be captured by Cdc7. During this docking process, the regulatory loop of Dbf4 occupies the activity center of the kinase to prevent premature substrate capturing by Cdc7. **e** DDK is docked onto each of the coupled MCM hexamers to function independently. **f** Conformational change in the kinase core and its subsequent dissociation from Mcm4 help to remove the regulatory loop of Dbf4 from Cdc7, enabling kinase center accessible to substrates. **g** After DDK phosphorylation, the NSD of Mcm4 becomes highly flexible, and exposes potential binding surfaces for origin firing factors to convert the DH into two active replisomes. **h** In the replisome, the binding surfaces of Dbf4 on Mcm4 and Mcm6 are covered by Tof1, which effectively redirects the DDK docked on the NTD-A of Mcm2 to other substrates. Note: the “replisome subcomplex” (CMG-Ctf4-Csm3-Tof1, EMD-10227) only depicts part of the replisome. **i** The DDK, recruited to the NTD-A of Mcm2 located at the front end of the translocating replisome, can extend its flexible kinase core to target substrates ahead of the replication fork.

Our DH-DDK structures also underscore the importance of the NTD-A subdomains from Mcm2, Mcm4 and Mcm6 as docking sites for DDK to assemble onto the DH. In fact, the NTD-As of various MCM subunits also serve as platforms for recruiting helicase-activating factors such as Cdc45 via the NTD-As of Mcm2 and Mcm5 and GINS via the NTD-As of Mcm5 and Mcm3 as well as Tof1 via the NTD-As of Mcm2, Mcm4, and Mcm6 (Supplementary Fig. 7). Although the overall structures are very similar among the six NTD-As, each NTD-A unit exhibits features specific for its binding partner(s) (Supplementary Fig. 7). As all MCM subunits have their own unique NTEs^48^, these flexible extensions may orchestrate the binding pattern for the helicase-activating factors to promote CMG formation and replisome assembly. Remarkably, though Tof1 and Dbf4 share the same binding surfaces on Mcm4 and Mcm6 (Supplementary Fig. 7b–d), their docking sites on the NTD-A of Mcm2 do not overlap (Supplementary Fig. 7i–l). This selection of docking sites may have important implications for the function of DDK in fork progression. During S phase, while the binding sites of Dbf4 on Mcm4 and Mcm6 are occupied by Tof1, DDK could still retain its association with the replisome mainly through its engagement with the NTD-A of Mcm2. With the help of Tof1^20^, DDK may target its substrates at the replication fork (Fig. 6). Indeed, DDK is required at the replication fork to coordinate DNA replication with other processes, such as DNA repair^18^ and meiotic recombination^20^.

Another striking feature of the DDK structure is the regulatory loop of Dbf4 embedded in the kinase center of Cdc7 to limit the accessibility of the activity center (Figs. 3a, c–e, and 4b). This special arrangement locks the kinase into an autoinhibitory state that effectively prevents premature capture of DDK substrates by Cdc7 to ensure a temporal order of DDK phosphorylation. A recent study reported that a similar autoinhibitory loop was also observed at the active center of CDK1 in the structure of CDK1-cyclin B1-CKS1 in complex with separase^49^. This recurring feature in CDK1 and DDK suggests that these cell cycle-dependent kinases may use a similar strategy to regulate substrate accessibility to their kinase active centers. Interestingly, this inhibition from Dbf4 regulatory loop is relieved when the kinase core disengages from Mcm4 and dislocates the inhibitory loop of Dbf4 from Cdc7 while remaining associated with Mcm4 through its downstream M4NB. Importantly, Thr506 and Ser507 from the regulatory loop of Dbf4 within the kinase center are situated very close to the ATP-γ-S nucleotide bound by Cdc7 (Fig. 3d, e). We propose that auto-phosphorylation of these sites by DDK may release the inhibitory loop of Dbf4 from the kinase center. This tantalising hypothesis merits further investigations.

In response to DNA damage in S phase, the checkpoint kinase Rad53 inhibits DNA replication initiation through targeting Dbf4, first, by phosphorylating Dbf4 to inhibit DDK kinase activity and, second, by binding to Dbf4 to keep DDK away from unfired origins^28,29^. Our DDK structure supports both of these independent mechanisms. So far, 26 potential Rad53 phosphorylation sites have been identified in Dbf4^28^. Four of them crucial for maintaining DDK activity under stress condition are clustered within the M4NB of Dbf4. We surmise that the inhibitory phosphorylation of this region by Rad53 might interfere with the binding of Dbf4 to the NTD-A of Mcm4. When these four sites were changed to alanine with key Rad53 phosphorylation sites in Sld3 also mutated, firing of late origins was observed in the mutant cells when challenged by hydroxyurea^28^. This result reinforces the importance of Dbf4-M4NB in displacing Mcm4-NSD to promote helicase activation. It has also been shown that the binding of Rad53 to DDK sterically hinders DDK from docking onto the DH independent of Rad53 kinase activity^30^. To understand the structural basis for this steric interference, we superimposed the DDK-DH structure with the crystal structure of Rad53-FHA1 bound to Dbf4-BRCT^50^ (Supplementary Fig. 7g, h). It appears that the FHA1 domain of Rad53 and the NTD-A of Mcm2 bind to different surfaces of the BRCT domain of Dbf4 and that Rad53 does not directly compete for the docking site on Dbf4-motif N for Mcm2. Rather, the bulkiness of the Rad53-DDK complex may block Dbf4 access to the NTD-A of Mcm2, or Rad53 may remodel DDK into a state incompetent for docking onto the DH.

In summary, this study unravels a multifaceted role of Dbf4 in modulating Cdc7 kinase activity on substrates of the DH (Fig. 6): (1) Dbf4 docks Cdc7 onto the DH; (2) Dbf4 situates the kinase core in a position to phosphorylate the NSD with prescribed priority; (3) Dbf4 displaces the NSD motif from its binding sites on Mcm4 to facilitate NSD phosphorylation by DDK; (4) the binding of Dbf4 to Cdc7 activates its kinase activity; and (5) Dbf4 regulates the accessibility of the kinase center to ensure an orderly targeting of its substrates. Furthermore, Dbf4 exerts another level of regulation for Cdc7 as a target of Rad53 to inhibit DDK activity upon DNA damage. Although the sequence homology of Dbf4 between yeast and human is limited, their structures appear very similar. Given the fact that the MCM proteins are highly conserved, we believe that human DDK would act on the human DH in a manner similar to its yeast counterpart for helicase activation. As DDK is an obvious target for cancer therapy, information about the unique features of Dbf4 in regulating Cdc7 activities provides the necessary details for developing novel anti-cancer drugs that selectively kill cancer cells with low toxic off-target side effects.

## Methods

### Yeast strains

All yeast strains used in this study were of the W303 (*leu2-3, -112 ura3-1 his3-11 trp1-1 ade2-1 can1-100*) genetic background. Mutagenesis was performed using PCR-based methods^51^. Proteins were purified from yeast strains containing integrated expression constructs (see Supplementary Table 3 for details). One-step PCR-based approach with pTF272 (pFA6a-TEV-6xGly-3xFlag-HphMX, Addgene) as DNA template was used to generate MCM7-TEV-3xFLAG or MCM2-TEV-3xFLAG tagging modification in the W303-1a background strain. Strain ySDK8 (a gift from J. Diffley) was used for DDK purification. Strains used for purification of DDK mutants were constructed by transforming ySDK8 with linearized expression vectors using standard genetic techniques (Supplementary Table 3). Strain used for purifying the mutant DHs was generated using one-step PCR based opproach with pYM-N7 (pFA6a-natNT-tADH1) as DNA template for N-terminal truncation of *MCM4* gene at its genomic locus.

### DH-DDK complex assembly

For each round of DH purification, 30 liters of log-phase G1 yeast cells were collected and cells were resuspended in 0.5 pellet volume of Benzonase Buffer (50 mM HEPES, pH = 7.6, 100 mM K-Glu, 1 mM EDTA, 2 mM NaF, 1 mM NaVO_3_, 8 mM MgCl_2_, 3 mM ATP, 1x protease inhibitor (Roche)). The suspensions were frozen drop-wise in liquid nitrogen before being processed in a Freezer/Mill^®^ High Capacity Cryogenic Grinder with 25 cycles at a crushing rate of 15. The resulting powders were thawed and resuspended in 3 volumes of Benzonase Buffer. The crude chromatin was then recovered and resuspended in Benzonase buffer for Benzonase (1U μl^−1^; Merck Biosciences 71206-3) treatment for 10 min at 37 °C to solubilize chromatin fractions. The suspension was then centrifuged for 20 min at 25,000 g. The clear phase was recovered, and subjected to anti-Flag immunoprecipitation with 1 ml bed volume of washed anti-Flag M2 agarose (Sigma, A2220) at 4 °C for 2 h. Beads were recovered, and washed extensively with benzonase buffer. For elution, we added 0.5 mg/ml (final concentration) 3×Flag peptide and incubated the beads at 4 °C for 25 min. The beads were washed once with an equal volume of benzonase buffer. Eluates were combined and concentrated using Amicon centrifugal filter concentrators (30-kDa molecular weight cutoff; Millipore). The concentrated complexes were then applied to the top of a 20–40% glycerol gradient in benzonase buffer with protease inhibitors. The gradients were centrifuged in a TLS-55 rotor (Beckman Optima TLX ultracentrifuge) at 105,000 *g* for 13 h. The fractions containing the DH were pooled and aliquoted for storage.

Full-length Dbf4-Cdc7 kinase complex was expressed and purified as described previously^10^ with the following modifications. Frozen cell powder was thawed completely on ice, resuspended in 1 volume of buffer A (25 mM Hepes-KOH pH 7.6, 0.05% NP-40, 10% Glycerol)/0.4 M NaCl/2 mM β-mercaptoethanol/protease inhibitors/phenylmethylfulfonyl fluoride (Thermo, 36978). The suspension was centrifuged for 32, 300 *g*, 4 °C, 1 h. The clear phase was recovered and subjected to Calmodulin affinity purification by adding 2 mM CaCl_2_ and 1.5 mL of packed beads of Calmodulin affinity resin (Agilent Technologies, 214303-52). After 3 h rotation at 4 °C, beads were collected, washed with 10 CVs of Buffer B (25 mM Hepes-KOH pH 7.6, 0.05% NP-40, 10% Glycerol, 0.1 mM EGTA, 0.1 mM EDTA)/0.4 M NaCl/2 mM CaCl_2_/2 mM β-mercaptoethanol. Calmodulin affinity resins were treated with lambda phosphatase (NEB, P0753L) for 1 h with rotation at 4 °C. Resin were washed with 5 CVs of Buffer B/0.4 M NaCl/2 mM CaCl_2_/2 mM β-mercaptoethanol. Elution was performed with Buffer B and subjected to fractionation over a Superdex 200 10/300 GL column (GE Healthcare) pre-equilibrated in Buffer B/0.4 M NaCl/2 mM β-mercaptoethanol. Fractions containing DDK were pooled and dialyzed against Buffer B/0.2 M K-Glutamate and aliquoted for storage.

For DH-DDK assembly without crosslinking, DDK (2.5 μM) was incubated in buffer (25 mM HEPES-KOH pH 7.6, 0.02% NP-40S, 150 mM K-Glutamate, 8 mM Mg(OAc)_2_, 2 mM β-mercaptoethanol, 1 mM ATP-γ-S) with the endogenously isolated MCM-DH (0.5μM) with either 1 mM of ATP-γ-S or 3 mM of ATP in a molar ratio of 10:1 on ice for 30 min before cryo-EM analyses. The actual amount of proteins used for the assembly was estimated using both SDS-PAGE and EM imaging.

To prepare sample with a mild crosslinking, the mixture of DDK and DH was applied on top of a 2-ml glycerol (20–40%) gradient in buffer (25 mM HEPES-KOH pH 7.6, 0.02% NP-40S, 150 mM KOAc, 8 mM Mg(OAc)_2_, 2 mM β-mercaptoethanol, 1 mM ATP-γ-S) containing glutaraldehyde (0–0.025%) for Grafix. The gradient was centrifuged in a Beckman TLS55 rotor (Beckman Optima TLX ultracentrifuge) for 13.5 h at 105,000 g at 4 °C. Fractions containing DH-DDK complexes were collected and the crosslinking reaction was quenched by addition of Tris-HCl (pH 8.0) buffer to a final concentration of 40 mM. Ultrafiltration for removal of glycerol and buffer exchange (25 mM HEPES-KOH pH 7.6, 0.02% NP-40S, 150 mM KOAc, 8 mM Mg(OAc)_2_, 2 mM β-mercaptoethanol, 1 mM ATP-γ-S) was performed with a centrifugal filter (Amicon Ultra-0.5 ml 50 k) at 6,000 g at 4 °C.

### Electron microscopy

For negative staining, the samples were stained with 2% uranyl acetate and examined using an FEI Tecnai T20 electron microscope operated at 120 kV to determine sample quality and estimate relative concentration of samples used for cryo-grid preparation (about 10 times higher).

For cryo-grid preparation, aliquots (4 μL) of samples were applied to glow-discharged C-flat or Quantifoil Au grids (R1.2/1.3, 400 mesh) and blotted using an FEI Vitrobot IV. Cryo grids were screened using an FEI Talos Arctica operated at 200 kV. High-quality grids were recovered and transferred to a Titan Krios microscope (FEI) operated at 300 kV for data collection. For DH-DDK samples without Grafix, images were collected using a GIF K2 camera (Gatan) with SerialEM 3.6.11^52^ in the super-resolution counting and movie mode, at a nominal magnification of 130,000× and defocus range of −1.0 to −3.0 μm. A total of 32 frames were collected for each micrograph, with dose rate at 10.2 e^−^ Å^−2^ s^−^ and a total exposure time of 6.4 s. For the crosslinked DH-DDK and the two mutant DH-DDK samples, images were collected using a GIF K3 camera (Gatan) in the super-resolution counting and movie mode, at a nominal magnification of 81,000× and defocus range of −1.0 to −2.5 μm. A total of 40 frames were collected for each micrograph, at a dose rate of 7.0 e^−^Å^−2^ s^−^ and a total exposure time of 6.5 s.

### Image processing

For the DH-DDK (ATP-γ-S) samples without cross-linking, two datasets were collected (4253 and 3644 movie stacks) and processed in similar procedures (Supplementary Fig. 1a, b). Drift-correction and electron dose-weighting were applied to the movie stacks using MotionCor2^53^, which generates summed images with or without dose weighting. Images without dose weighting were used to evaluate the parameters of contrast transfer function (CTF) by CTFFIND4 v4.1.13^54^. Summed images and CTF power spectra were screened manually to select high-quality images for further processing. For each dataset, after 2D and global 3D classifications using RELION3.1^55^, high-quality particles were selected and subjected to one round of 3D refinement with C2 symmetry imposed. The particles were then duplicated using the command “relion_particle_symmetry_expand” in RELION3.1 and a mask-based 3D classification (on DDK region) with “--skip alignment” option (also with particles re-centered at DDK region) was performed. According to DDK occupancy in the resulting 3D classification maps, particles with and without DDK bound were estimated to be 7.9% and 92.1%, respectively (Supplementary Fig. 1a). Particles without DDK bound (empty DH) were pooled and re-centered at Mcm4-NSD region, and subjected to another round of focused 3D classification. Those classes with Mcm4-NSD stably anchored to Mcm4-NTD from both datasets ofthe DH-DDK (ATP-γ-S) samples were combined and refined to generate a global density map for the DDK-free DH at an overall resolution of 3.2 Å (Supplementary Fig. 1a, b).

The dataset of the DH-DDK (ATP) samples (3055 movie stacks) was similarly processed (Supplementary Fig. 1c). From the DDK-free DH particles (90.3% of all particles), focused classification on Mcm4-NSD region did not reveal any class with structured Mcm4-NSD.

For the mutant DH-DDK (ATP-γ-S) samples, 3938 and 4027 movie stacks were collected for the DHΔN140-DDK and DHΔN174-DDK datasets respectively. They were also similarly processed as the WT DH-DDK datasets (Supplementary Fig. 1d, e). After the first round of 3D classification, DDK-bound particles were selected for a second round of focused 3D classification to improve the occupancy and conformational homogeneity of DDK region, and the final maps for the DH-DDK complexes were refined to 3.8 Å and 3.9 Å for the two datasets respectively. The DDK-free particles from the DHΔN140-DDK dataset were subjected to another round of focused 3D classificastion on the Mcm4-NSD region. A class of particles containing density of truncated NSD was clearly separated, which was refined to a global resolution of 3.8 Å (Supplementary Fig. 1d, f).

For the crosslinked DH-DDK (ATP-γ-S) samples, a total of 5184 qualified movie stacks were selected for image processing (Supplementary Fig. 2). Around 1461 K particles were auto-picked, and screened by a cascade of 2D and global 3D classifications, resulting in a dataset of 679 K particles for further processing. Similar as non-crosslinked samples, particles were first refined with C2 symmetry imposed. Next, after C2 symmetry expansion, particles were re-centered at DDK region and subjected to one round of focused classification on DDK region. Among the five resulting classes, two of them display stable binding of both Dbf4 and Cdc7. These two classes (208 K particles) were combined for high-resolution refinement using dose-weighted particles. After CTF refinement and Bayesian polishing, the final density map for a stable DH-DDK complex (State I) was resolved at an overall resolution of 2.9 Å (Supplementary Fig. 2d, f). Further refinement of the kinase core region of DDK using density substraction improved the resolution of the kinase core region to 3.8 Å (Supplementary Fig. 2d, f). To investigate other possible conformations of DDK on the DH, two classes from the first round focused classification were combined (both only display stable binding for the N-terminal half of Dbf4) for another round of focused classification, from which three representative conformations (State II to IV) for DDK were identified.

For ease of data presentation, we also divided the particles of the crosslinked DH-DDK into six groups according to the results from the first round of focused 3D classification (Supplementary Figs. 2d and 3). The grouping standards were based on the occupancy of the two symmetrical DDK-binding sites and the general stability of DDK (Stable DDK vs Stable Dbf4-NTD). Groups I, II, and III represent the DHs with both sites occupied; Groups IV and V represent the DHs with one DDK site occupied, and Group VI represents the DHs free of DDK binding. These groups of particles were refined to resolutions of 3.3–4.0 Å, representing major compositional and conformational populations of DH-DDK in the reaction.

The resolution estimation was based on gold-standard FSC at the cutoff of 0.143. The local resolution maps were generated using ResMap^56^. Structural analysis was done with UCSF Chimera 1.11.2^57^ and Pymol (http://pymol.org).

### Model building

Modeling was performed using the DH-DDK maps from crosslinked datasets. The initial templates used for modeling were from a 2.5 Å-resolution cryo-EM structure of the yeast DH (PDB: 7W8G) generated with the free DH particles from the non-crosslinking DH-DDK sample treated with ATP-γ-S (Supplementary Table 1), crystal structures of human DDK (PDB: 6YA7) and yeast Dbf4 BRCT domain (PDB: 5T2F). Each domain of the DH-DDK subunits was manually fitted into the density map in Chimera, followed by manual rebuilding using Coot 0.9.5^58^. Secondary structure prediction of Cdc7 and Dbf4 subunits was performed using PSIPRED^59^. The nucleotide moieties in the nucleotide-binding pockets of MCM subunits were modeled as ATP-γ-S or ADP. The models were refined against the 2.9-Å density map and 3.8-Å local density map of DDK region using Phenix.real_space_refine^60^ with secondary structure restraints and geometry restraints imposed. The final models were evaluated by MolProbity 1.19.2^61^, and statistics are presented in Supplementary Table 1.

### Kinase assays

For in vitro kinase assay, both WT and mutant DDKs and DH were incubated on ice in kinase buffer (50 mM HEPES pH7.5, 100 mM K-Glutamate, 1 mM EDTA, 8 mM MgCl_2_, 3 mM ATP, 4 mM NaF, 2 mM NaVO_3_), and 1x protease inhibitor. Reactions were terminated at indicated time points by adding 2x Laemmli sample buffer and then boiled immediately at 100 °C for 5 min followed by SDS-PAGE (6%) and western blotting analysis. DDK autophosphorylation was performed with both WT and mutant DDK preparations in kinase buffer at 30 °C for 30 min, and the protein samples were analyzed by SDS-PAGE (7.5%) and visualized by Coomassie blue staining.

### Quantification and statistical analysis

The orientation distribution of the particles used in the final reconstruction was calculated using RELION 3.1^55^. The local resolution map was calculated using ResMap v1.1.4^56^.

### Reporting summary

Further information on research design is available in the Nature Research Reporting Summary linked to this article.

## Supplementary information

Supplementary Information

Description of Additional Supplementary Files

Supplementary Movie 1

Supplementary Movie 2

Reporting Summary

## Data Availability

The data that support this study are available from the corresponding authors upon reasonable request. Cryo-EM maps of free DH in ATP-γ-S and ATP, DH-DDK State I-II, DDK kinase core, Dbf4-NTD+Mcm2-NTD-A, DH-DDK Group I-V, DHΔN140-DDK, DHΔN174-DDK, DHΔN140, DHΔN174, and DH have been deposited in the Electron Microscopy Data Bank (EMDB) with accession codes EMD-31684, EMD-31701, EMD-31685, EMD-31696, EMD-31686, EMD-31687, EMD-31688, EMD-31689, EMD-31690, EMD-31691, EMD-31692, EMD-31694, EMD-31695, EMD-31699, EMD-31700 and EMD-32355 respectively. Atomic coordinates of free DH in ATP-γ-S, DH-DDK Group I and DH have been deposited in the Protein Data Bank (PDB) with accession code 7V3U, 7V3V and 7W8G respectively. Source data are provided with this paper.

## Source data

Source Data
