# Author Correction: Structural Insight into the MCM double hexamer activation by Dbf4-Cdc7 kinase

**DOI:** 10.1038/s41467-022-30188-9

**Published:** 2022-04-27

**Authors:** Jiaxuan Cheng, Ningning Li, Yunjing Huo, Shangyu Dang, Bik-Kwoon Tye, Ning Gao, Yuanliang Zhai

**Affiliations:** 1 State Key Laboratory of Membrane Biology, Peking-Tsinghua Joint Center for Life Sciences, School of Life Sciences, Peking University, Beijing, 100871 China; 2 School of Biological Sciences, The University of Hong Kong, Hong Kong, China; 3 Division of Life Science, The Hong Kong University of Science & Technology, Hong Kong, China; 4 Institute for Advanced Study, The Hong Kong University of Science & Technology, Hong Kong, China; 5 Department of Molecular Biology & Genetics, Cornell University, Ithaca, NY 14853 USA; 6 National Biomedical Imaging Center, Peking University, Beijing, 100871 China; 7 Present Address: Vaccine Research Center, National Institute of Allergy and Infectious Diseases, National Institutes of Health, Bethesda, MD USA

**Keywords:** Origin firing, Cryoelectron microscopy, Enzyme mechanisms

Correction to: *Nature Communications* 10.1038/s41467-022-29070-5, published online 16 March 2022.

The original version of this Article contained an error in Data Availability statement, which incorrectly indicated two of the Electron Microscopy Data Bank (EMDB) accession codes: EMD-31697 and EMD-31670.

The correct version replaces:

EMD-31697 with EMD-31687 and

EMD-31670 with EMD-31700.

This has been corrected in both the PDF and HTML versions of the Article.

