# Supplementary material for: Structural Insight into the MCM double hexamer activation by Dbf4-Cdc7 kinase
Source: Nat Commun. 2022 Mar 16;13:1396. doi: 10.1038/s41467-022-29070-5 (PMC8927117; doi:10.1038/s41467-022-29070-5)
Supplement: Supplementary file 1 — Supplementary Information [file 41467_2022_29070_MOESM1_ESM.pdf]

Supplementary Information

Structural Insight into the MCM Double Hexamer Activation by Dbf4-Cdc7 Kinase

Jiaxuan Cheng[1#], Ningning Li[1#], Yunjing Huo[2], Shangyu Dang[3], Bik-Kwoon Tye[4,5]*, Ning Gao[1]* & Yuanliang Zhai[2]*

[#]These authors contributed equally to this work

*Co-corresponding authors

This document contains 7 Supplementary Figures and 3 Supplementary Tables.

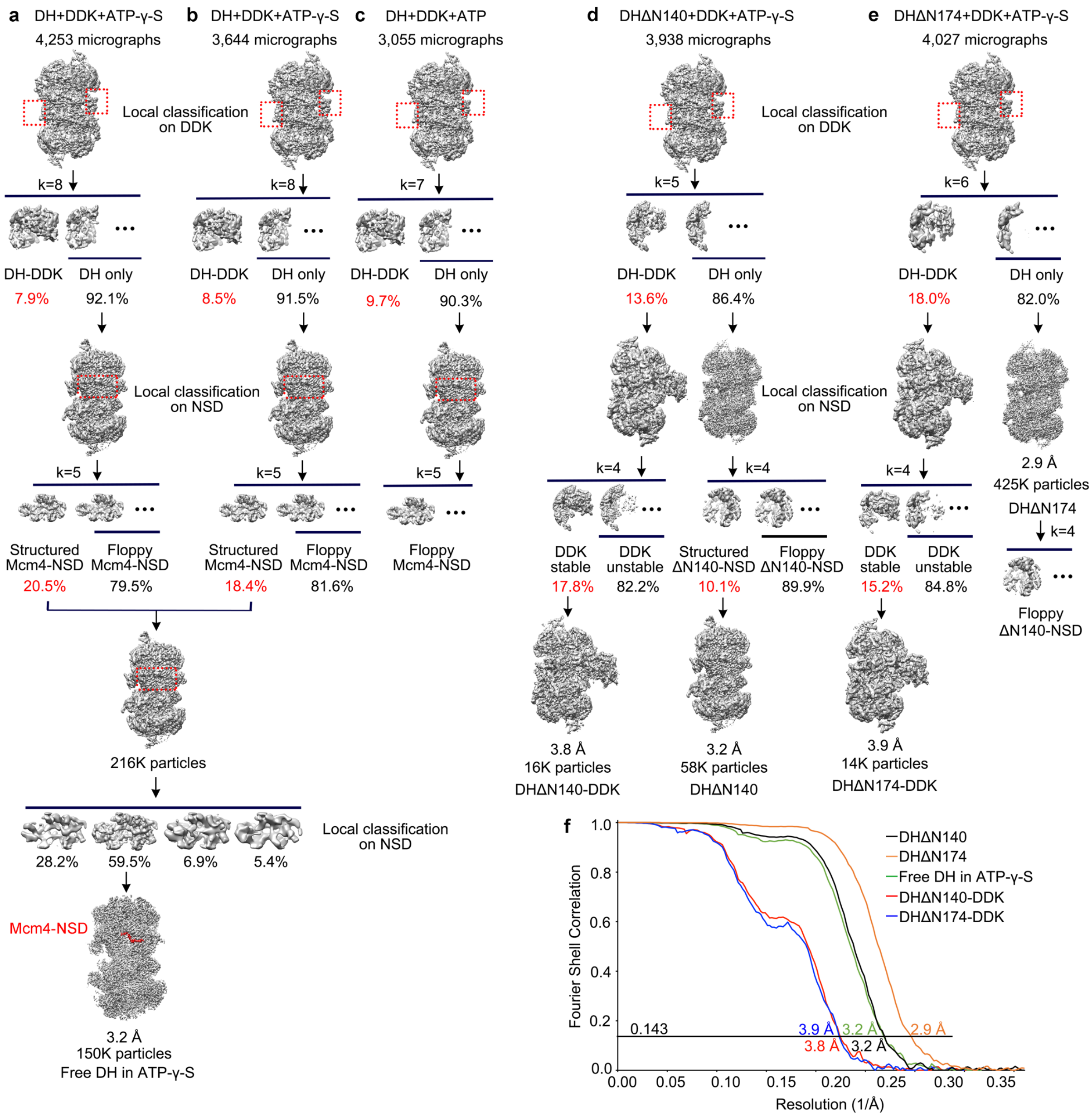

Supplementary Figure 1. Image processing of the DH-DDK related samples

a-e, Workflow of image processing for the DH-DDK samples treated with either ATP-γ-S (**a** and **b**) or ATP (**c**) and the mutant DH-DDK samples including DHΔN140 (**d**) and DHΔN174 (**e**). See Materials and Methods for details. Crosslinking was not applied to these samples. Notably, structures (major populations) of the DH-DDK treated with either ATP-γ-S or ATP are almost identical to the State I structure of the DH-DDK (ATP-γ-S) stabilized by crosslinking. **f**, FSC curves of the final density maps for the indicated complexes.

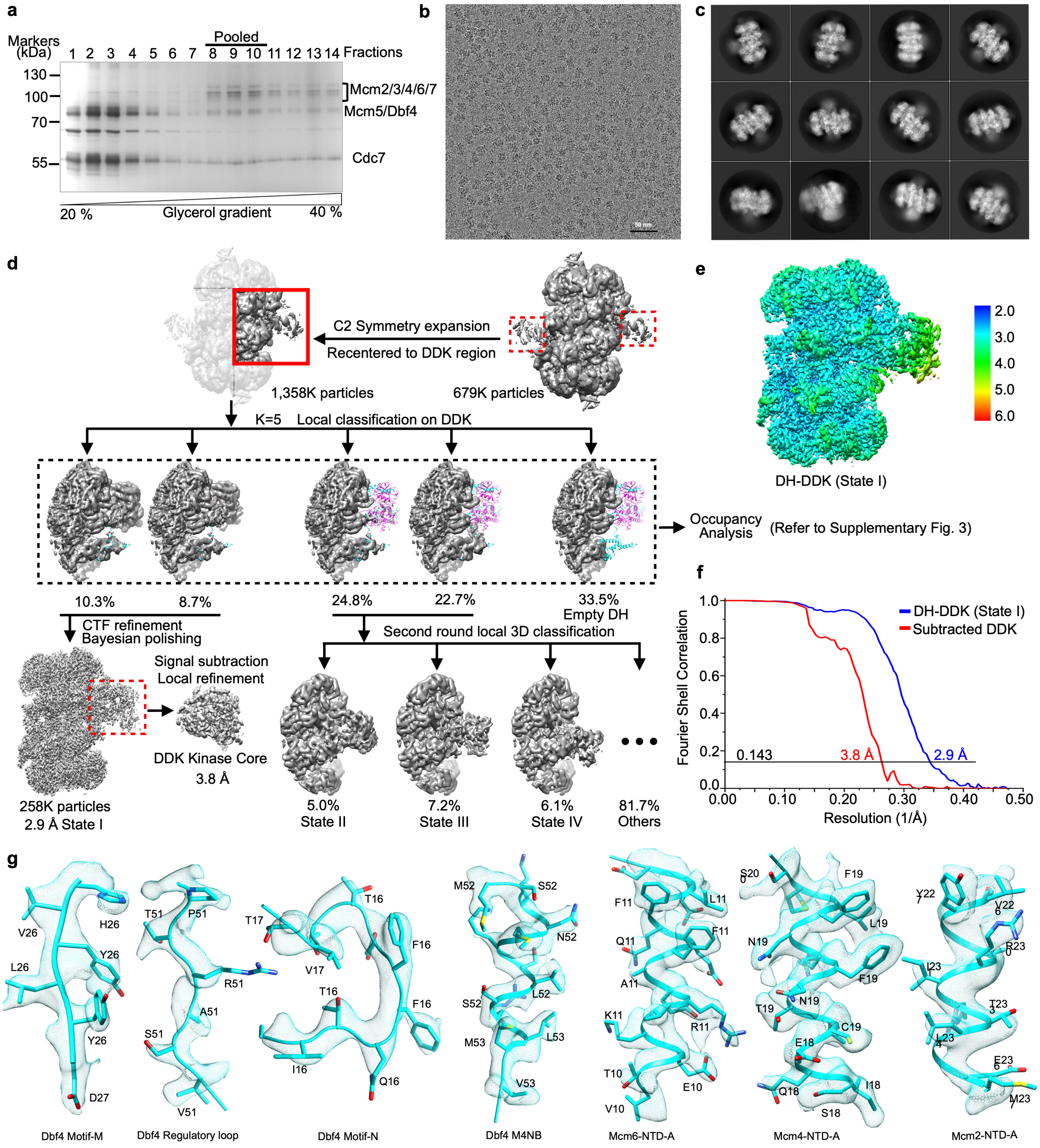

Supplementary Figure 2. Image processing of the crosslinked DDK-DH sample
a, SDS-PAGE analysis of the glycerol gradient fractions. The mixture of DDK and DH (no fixation) was subjected to 20-40% glycerol gradient centrifugation. Fractions were collected, resolved on SDS-PAGE and visualized by silver staining. This analysis showed that peak fractions (8-10) contain intact DH-DDK complexes. A biological replicate of the experiment was performed with similar result. Based on this result, similar fractions containing crosslinked samples after grafix were pooled and processed for further EM analysis. **b**, A representative raw cryo-EM image of the crosslinked DH-DDK sample. A total of 5,184 raw micrographs were selected for data processing. **c**, 2D class averages of the crosslinked DH-DDK sample.
d, Workflow of image processing of the DH-DDK particles. See Materials and Methods for details. During the second round of local 3D classification, the particles were split into 15

classes. We identified three States (II-IV, accounting for 18.3% of the subset II particles) of DDK on the DH that exhibit relatively stable kinase core of DDK residing in different wobbling positions. The remaining 81.7% particles accounting for 12 classes (others, not shown). DDK densities in these classes are clearly present, but more fragmented, indicating that DDK likely has a continuous movement on the DH. **e**, The final density map of the DH-DDK (State I) color-coded to indicate the range of local resolution. **f**, FSC curves of the final density maps for the indicated complexes. **g**, Local densities of representative regions for Dbf4 and NTD-As of Mcm2/4/6 from the final cryo-EM density map of the DH-DDK (State I).

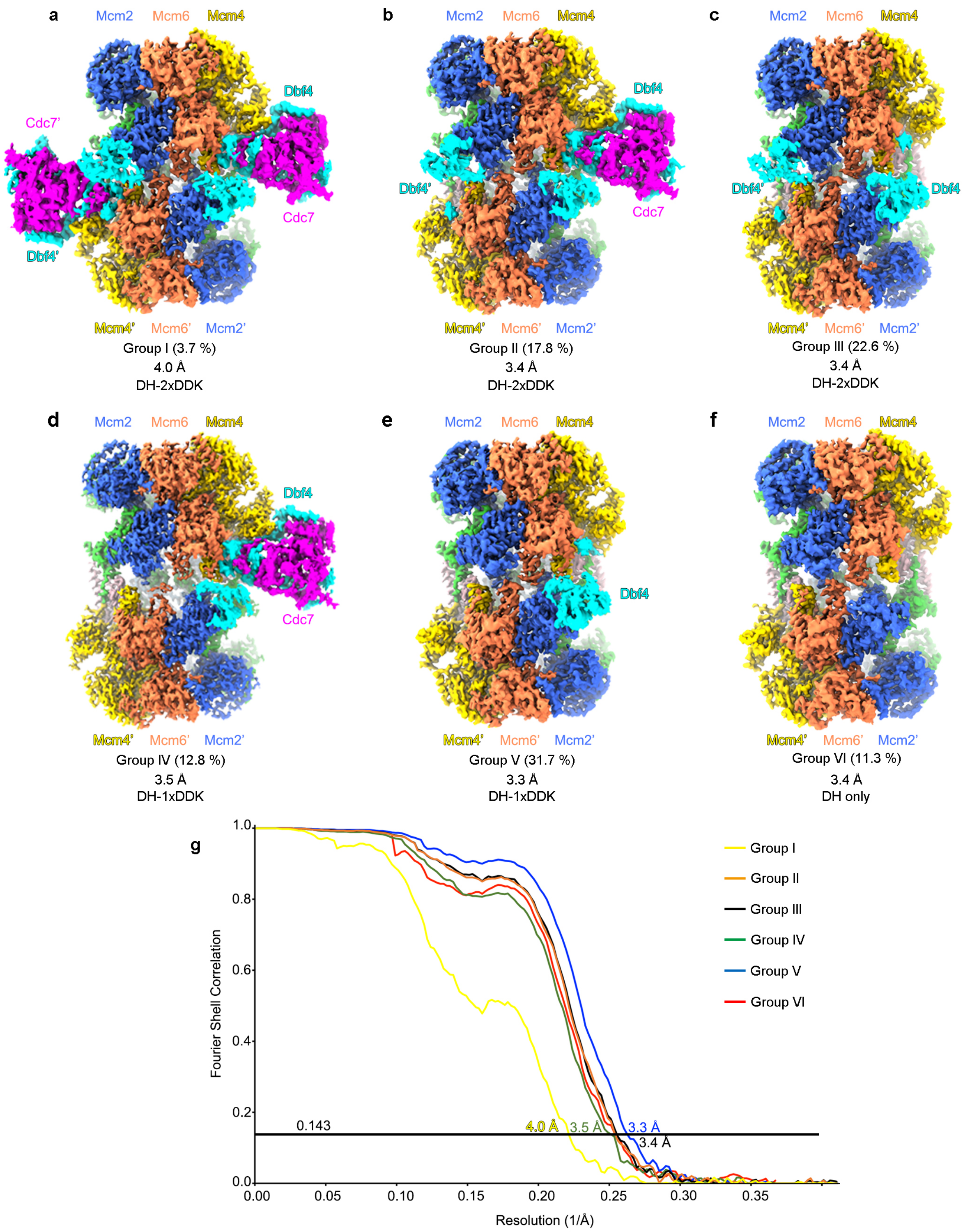

Supplementary Figure 3. Stochastic binding of DDK to the DH.
a-c, Side views of the segmented cryo-EM density maps of the DH bound with two DDKs (Groups I-III). These maps differ primarily in the conformation of kinase core. **d-e**, Side views of the segmented cryo-EM density maps of the DH bound with one DDK (Groups IV-V). **f**, Side views of the cryo-EM density maps of the free DH (Group VI). **g**, FSC curves of the density maps of Groups I-VI.

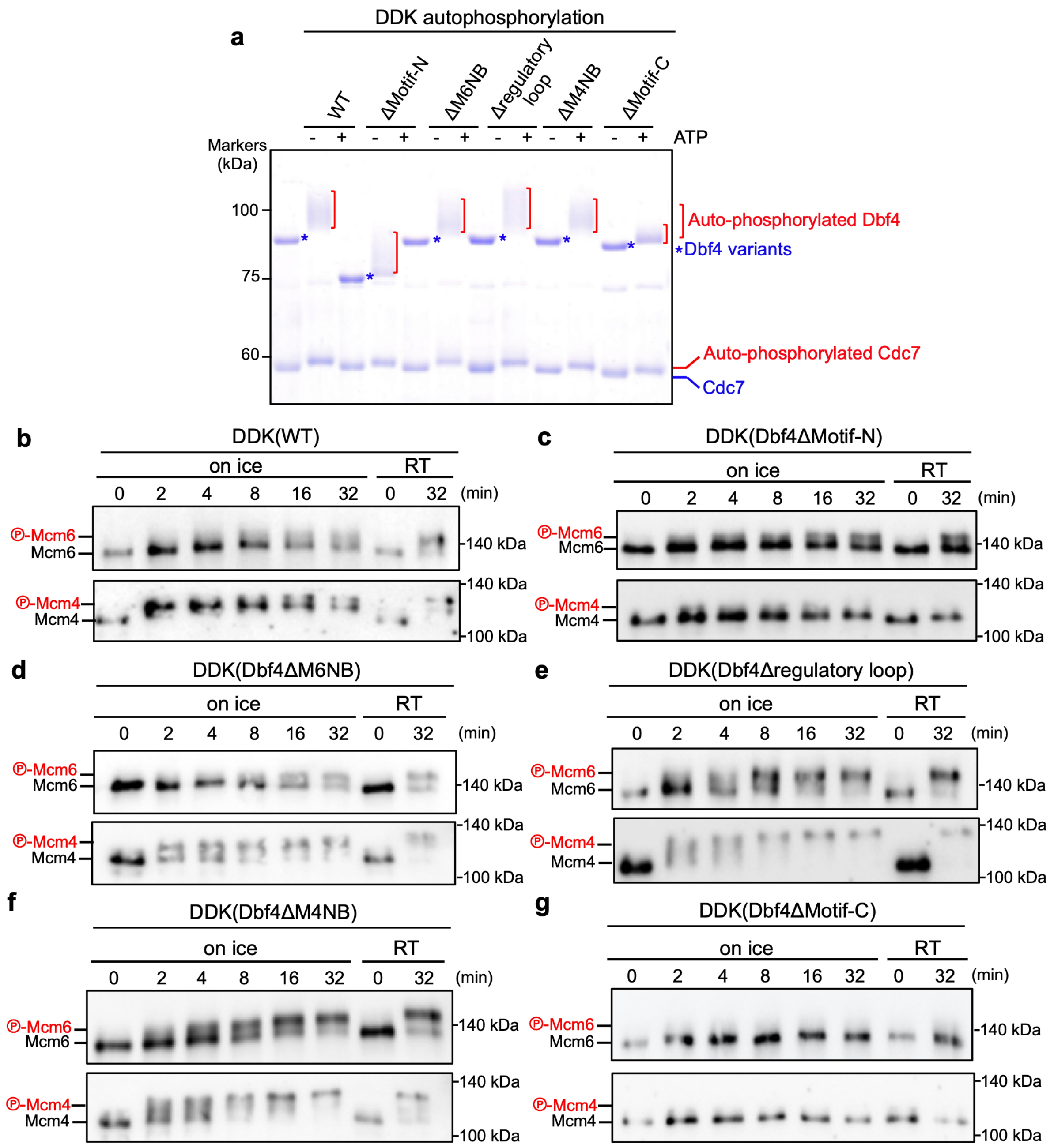

Supplementary Figure 4. Kinase activities of various DDK constructs toward MCM-DH. **a**, SDS-PAGE (7.5% gel) analysis of the WT and mutant DDK preparations and their associated kinase activities through in vitro DDK autophosphorylation assay. The indicated bands were visualized by Coomassie blue staining. **b-g**, The kinase reactions with MCM-DH and relevant DDKs were conducted on ice (left lanes) or at room temperature (RT, two right lanes) at indicated time points and analyzed by SDS-PAGE (6 % gel) and immunoblotting of Mcm4 and Mcm6. Ⓟ represents the phosphorylated form of the relevant MCM subunit. A biological replicate of each experiment was performed with similar results.

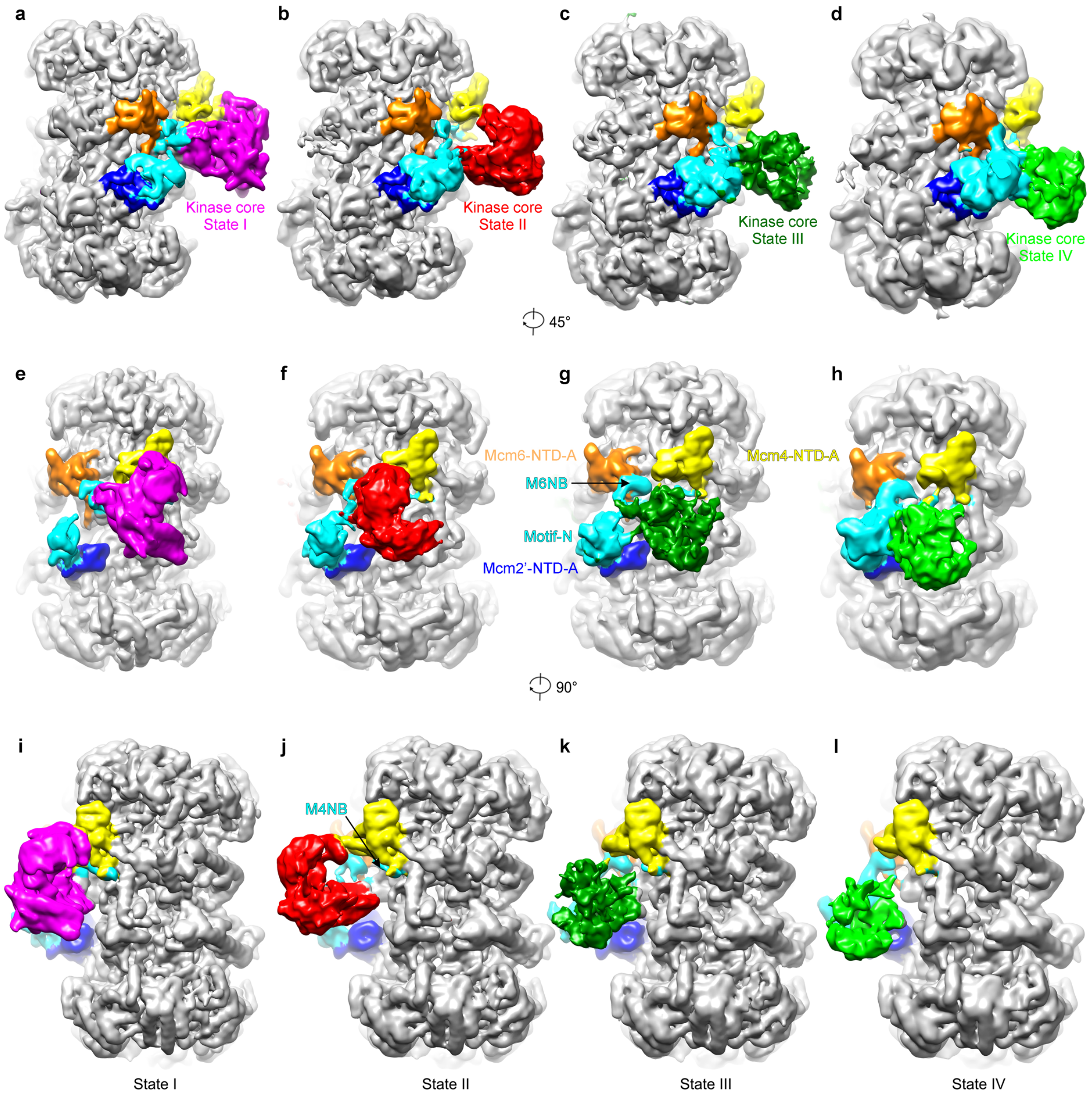

Supplementary Figure 5. Wobbling conformations of the kinase core of DDK on the DH. a-d, Comparison of the conformations of the kinase core from the different states (I-IV) of the DH-DDK complex. Density maps of the kinase core region and the NTD-As of Mcm2', Mcm6 and Mcm4 are color-coded and labelled as indicated. **e-h**, Same as **a-d** but shown with a 45° rotation along the cylinder axis. **i-l**, Same as **e-h** but shown with a further 90° rotation.

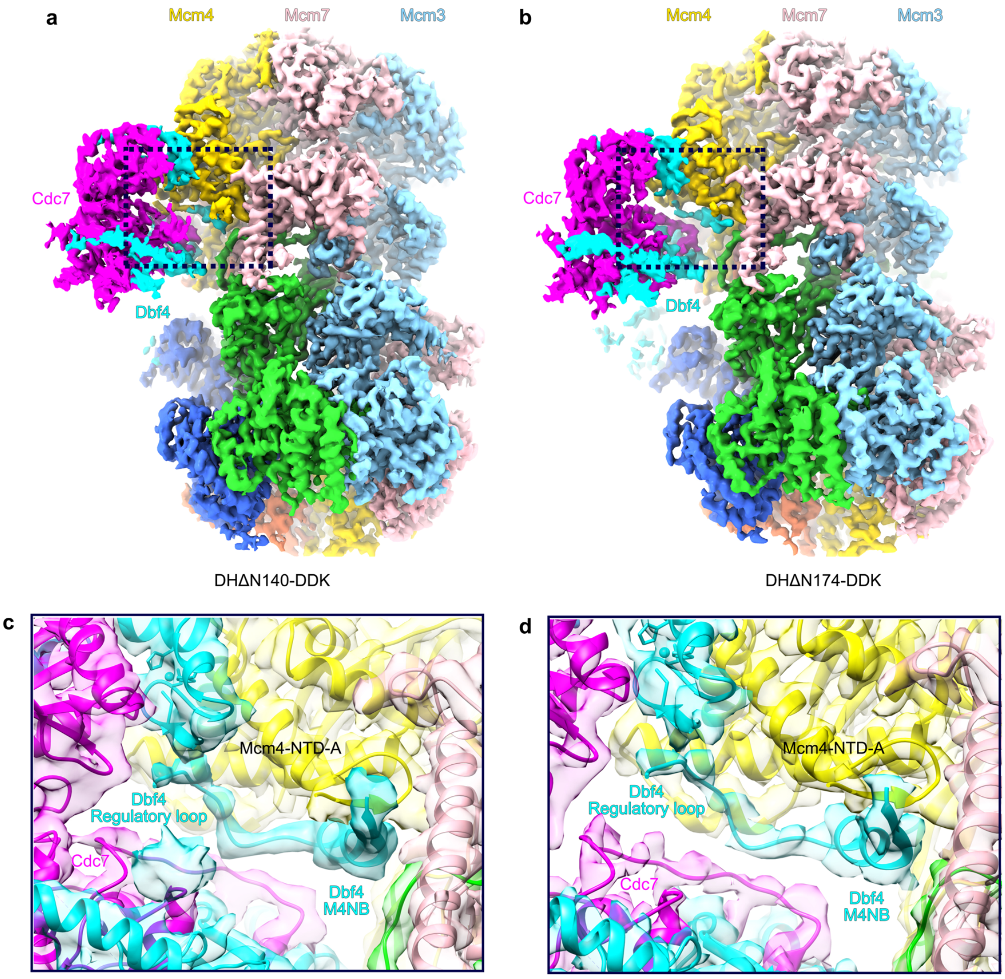

Supplementary Figure 6. DDK docking onto the mutant DHs.

a, b Side views of the cryo-EM maps of the DHΔN140-DDK (**a**) and DHΔN174-DDK (**b**), highlighting the kinase core of DDK engaging with the NTD-A of Mcm4.

c, d Magnified views of the boxed regions in (**a**) and (**b**) respectively with the atomic model superimposed. Note that the conformations of the M4NB and regulatory loop of Dbf4 in these mutants are almost identical as those in the WT DH-DDK (State I), and the NSD motif is not required for DDK docking onto the NTD-A of Mcm4.

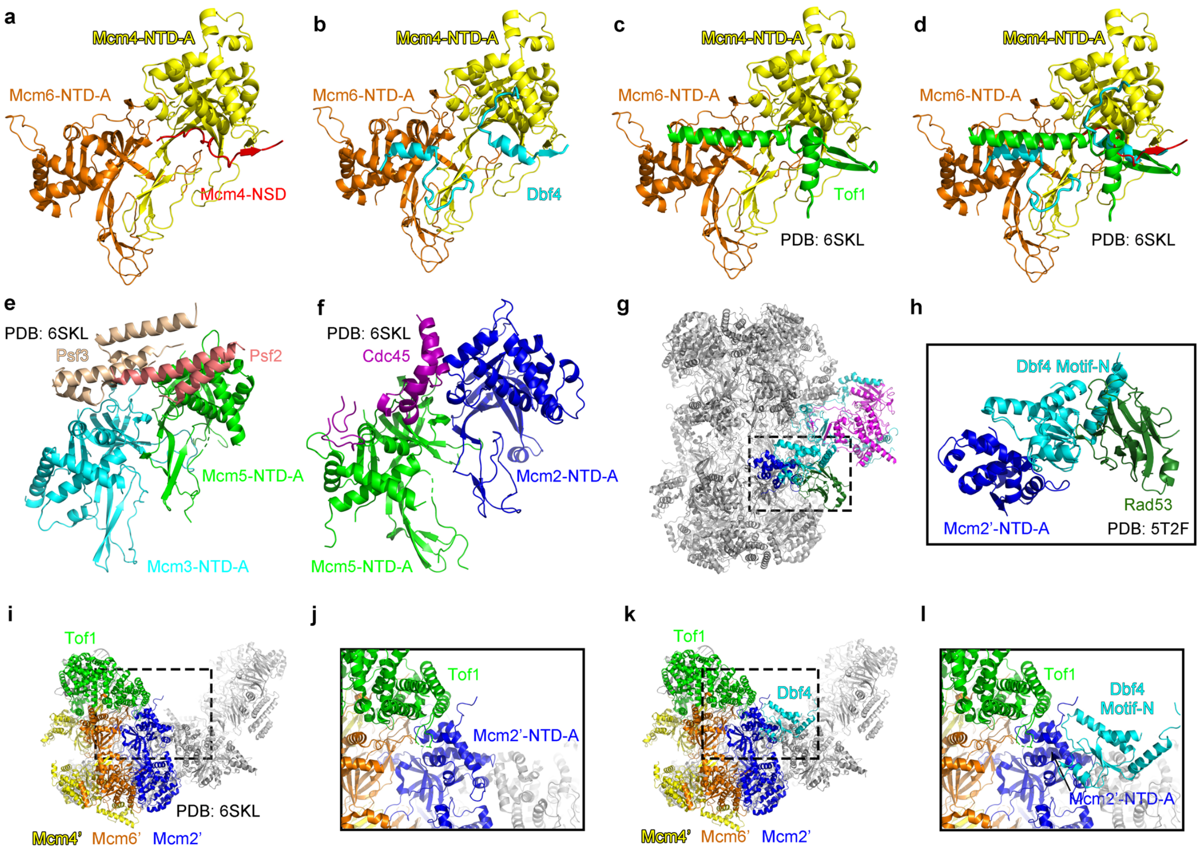

Supplementary Figure 7. The NTD-As of MCM subunits serving as distinct docking sites for various replication factors.

a, Mcm4-NSD (red) is nested on the NTD-A of Mcm4 (yellow). **b**, M6NB and M4NB of Dbf4 (cyan) are anchored on the NTD-As of Mcm6 (orange) and Mcm4 (yellow) respectively. **c**, Tof1 (green) associates with the NTD-As of Mcm6 and Mcm4. For clarity, only the indicated regions of the replisome structure (PDB code 6SKL) are shown. **d**, Superimposition of the structures of DH, DH-DDK and replisome (PDB code 6SKL) using DH as a reference. Note that Mcm4-NSD, Dbf4-M4NB and Tof1 share the same binding surface on the NTD-A of Mcm4, and the binding site of Dbf4-M6NB on the NTD-A of Mcm6 overlaps that of Tof1.**e**, Binding surfaces of GINS on the NTD-As of Mcm3 and Mcm5 (PDB code 6SKL). **f**, Binding surfaces of Cdc45 on the NTD-As of Mcm2 and Mcm5 (PDB code 6SKL). **g**, Superimposition of the structures of DH-DDK and Dbf4-Motif N-Rad53-FHA (PDB code 5T2F) using Dbf4 Motif-N as a reference. **h**, Magnified view of the boxed region in (**g**). Note that different interfaces of Dbf4 motif N engage with Mcm2-NTD-A and Rad53-FHA. **i**, Side view of the replisome structure (PDB code 6SKL) displayed in cartoon, highlighting the NTD-A of Mcm2 as the only potential binding site for DDK on replisome. **j** Magnified view of the boxed region in (**i**). **k**, Superimposition of the replisome structure (PDB code 6SKL) with the DH-DDK using Mcm2-NTD-A as a reference. Note that the binding site of Dbf4 motif N on the NTD-A of Mcm2 does not overlap with Tof1. **l**, Magnified view of the boxed region in (**k**).

Supplementary Table 1 Summary of Cryo-EM Data Collection and Model Refinement

	High-resolution DH (EMD-32355) (PDB 7W8G)	Free DH in ATP-γ-S with structured Mcm4-NSD (EMDB-31684) (PDB: 7V3U)	DH-DDK (State I) (EMDB-31685) (PDB: 7V3V)	DDK Kinase Core (EMDB-31686)	Dbf4-NTD+ Mcm2-NTD-A (EMDB-31687)
**Data collection and processing**					
Magnification	130,000X	130,000X		81,000X	
Voltage (kV)	300	300		300	
Electron exposure (e–/Å^2)	46/dose weighting	46/dose weighting		46/dose weighting	
Defocus range (μm)	1-3.5	1-3.5		1-3.5	
Pixel size (Å)	1.052	1.052		1.06	
Symmetry imposed	C2	C1		C1	
Initial particle images (no.)	973K	216K		679K	
Final particle images (no.)	576K	150K		258K	
Map resolution (Å)	2.52	3.2	2.9	3.8	4.0
FSC threshold	0.143	0.143	0.143	0.143	0.143
**Refinement**					
Initial model used (PDB code)	3JA8	7W8G	7W8G 6YA7 5T2F		
Map sharpening *B* factor (Å^2)	-60	-80	-50	-136	-200
Model composition					
Non-hydrogen atoms	62472	62658	68358		
Protein residues	7878	7900	8587		
Ligands					
Nucleotides	12	12	13		
Mg^{2+}	12	12	12		
Zn^{2+}	10	10	11		
R.m.s. deviations					
Bond lengths (Å)	0.0039	0.0042	0.0042		
Bond angles (°)	0.79	0.71	0.82		
Validation					
MolProbity score	1.35	1.38	1.47		
Clashscore	5.65	5.25	6.23		
Poor rotamers (%)	0.04	0.03	0.07		
Ramachandran plot					
Favored (%)	97.83	97.52	97.31		
Allowed (%)	2.16	2.48	2.64		
Disallowed (%)	0.01	0.00	0.05		

Supplementary Table 2 A summary of the structures determined in this study

PDB and EMD Codes	PDB entry title
PDB-7V3U<br>EMD-31684	Cryo-EM structure of MCM double hexamer with structured Mcm4-NSD
EMD-31701	Cryo-EM structure of MCM double hexamer phosphorylated by DDK
PDB-7V3V<br>EMD-31685	Cryo-EM structure of MCM double hexamer bound with DDK in State I
EMD-31696	Cryo-EM structure of MCM double hexamer bound with DDK in State II
EMD-31688	Cryo-EM structure of MCM double hexamer bound with two DDKs (Group I)
EMD-31689	Cryo-EM structure of MCM double hexamer bound with two DDKs (Group II)
EMD-31690	Cryo-EM structure of MCM double hexamer bound with two DDKs (Group III)
EMD-31691	Cryo-EM structure of MCM double hexamer bound with one DDK (Group IV)
EMD-31692	Cryo-EM structure of MCM double hexamer bound with one DDK (Group V)
EMD-31699	Cryo-EM structure of mutant MCM double hexamer (Mcm4ΔN140)
EMD-31700	Cryo-EM structure of mutant MCM double hexamer (Mcm4ΔN174)
EMD-31694	Cryo-EM structure of mutant MCM double hexamer (Mcm4Δ140) bound with DDK
EMD-31695	Cryo-EM structure of mutant MCM double hexamer (Mcm4ΔN174) bound with DDK (Δ174)
EMD-31686	Cryo-EM map of DDK subtracted from DH-DDK complex
EMD-31697	Cryo-EM map of Dbf4-NTD engaged with Mcm2-NTD-A subtracted from the DH-DDK complex
PDB-7W8G<br>EMD-32355	Cryo-EM structure of MCM double hexamer

Supplementary Table 3 Yeast strains used in this study

Strain	Genotype	Source
304	*MATa ade2-1 trp1-1 leu2-3,112 his3- 11,15 ura3-1 can1-100 bar1Δ::natNT*	This study
305	*MATa ade2-1 trp1-1 leu2-3,112 his3- 11,15 ura3-1 can1-100 bar1Δ::natNT MCM7-3xFlag-phpNT1*	This study
ySDK	*MATa ade2-1 ura3-1 his3-11,15 trp1-1 leu2-3,112 can1-100, pep4 Δ::KanMX, trp1::TRP1pRS304CDC7, CBP-DBF4*	Diffley lab (On et al., 2014)
306	*MATa ade2-1 trp1-1 leu2-3,112 his3- 11,15 ura3-1 can1-100 bar1Δ::natNT MCM7-TEV-3xFlag-phpNT1 mcm4::KanMX-ADH-mcm4 ΔN2-140*	This study
307	*MATa ade2-1 trp1-1 leu2-3,112 his3- 11,15 ura3-1 can1-100 bar1Δ::natNT MCM7-TEV-3xFlag-phpNT1 mcm4::KanMX-ADH-mcm4 ΔN2-174*	This study
308	*MATa ade2-1 ura3-1 his3-11,15 trp1-1 leu2-3,112 can1-100, pep4 Δ::KanMX, trp1::TRP1pRS304CDC7, CBP-DBF4, leu2::LEU2pRS405 3xFlag-DBF4*	This study
309	*MATa ade2-1 ura3-1 his3-11,15 trp1-1 leu2-3,112 can1-100, pep4 Δ::KanMX, trp1::TRP1pRS304CDC7, CBP-DBF4, leu2::LEU2pRS405 3xFlag-DBF4Δ105-221*	This study
310	*MATa ade2-1 ura3-1 his3-11,15 trp1-1 leu2-3,112 can1-100, pep4 Δ::KanMX, trp1::TRP1pRS304CDC7, CBP-DBF4, leu2::LEU2pRS405 3xFlag-DBF4Δ231-259*	This study
311	*MATa ade2-1 ura3-1 his3-11,15 trp1-1 leu2-3,112 can1-100, pep4 Δ::KanMX, trp1::TRP1pRS304CDC7, CBP-DBF4, leu2::LEU2pRS405 3xFlag-DBF4Δ500-515*	This study
312	*MATa ade2-1 ura3-1 his3-11,15 trp1-1 leu2-3,112 can1-100, pep4 Δ::KanMX, trp1::TRP1pRS304CDC7, CBP-DBF4, leu2::LEU2pRS405 3xFlag-DBF4Δ516-534*	This study
