# Supplementary material for: Structural Insight into the MCM double hexamer activation by Dbf4-Cdc7 kinase
Source: Nat Commun. 2022 Mar 16;13:1396. doi: 10.1038/s41467-022-29070-5 (PMC8927117; doi:10.1038/s41467-022-29070-5)
Supplement: Supplementary file 2 — Description of Additional Supplementary Files [file 41467_2022_29070_MOESM2_ESM.pdf]

Description of Additional Supplementary Files

File name: Supplementary Movie 1

Description: **Overall structure of the DH-DDK complex and arrangement of various motifs and domains of Dbf4.** The cryo-EM density map of the DH-DDK superimposed with atomic model is first shown in a continuous rotation around cylindrical axis, highlighting the locations of Dbf4 and Cdc7 subunits in the structure. Subsequently, the interfaces between Dbf4 and the NTD-As of Mcm2'/6/4 and the regulatory loop of Dbf4 are shown in zoom-in views.

File name: Supplementary Movie 2

Description: **Dynamic kinase core of DDK on the DH.** The DH-DDK structure (State I) in cartoon view morphs into its State II structure, highlighting the conformational changes that occur in the kinase core region of DDK between these two states.
