# Supplementary material for: Structural Insight into the MCM double hexamer activation by Dbf4-Cdc7 kinase
Source: Nat Commun. 2022 Mar 16;13:1396. doi: 10.1038/s41467-022-29070-5 (PMC8927117; doi:10.1038/s41467-022-29070-5)
Supplement: Supplementary file 5 — Reporting Summary [file 41467_2022_29070_MOESM5_ESM.pdf]

nature research

Corresponding author(s): Bik Kwoon Tye
Ning Gao
Yuanliang Zhai

Last updated by author(s): 05/02/2022

Reporting Summary

Nature Research wishes to improve the reproducibility of the work that we publish. This form provides structure for consistency and transparency in reporting. For further information on Nature Research policies, see our Editorial Policies and the Editorial Policy Checklist.

Statistics

For all statistical analyses, confirm that the following items are present in the figure legend, table legend, main text, or Methods section.

n/a	Confirmed	
☐	☒	The exact sample size (*n*) for each experimental group/condition, given as a discrete number and unit of measurement
☐	☒	A statement on whether measurements were taken from distinct samples or whether the same sample was measured repeatedly
☒	☐	The statistical test(s) used AND whether they are one- or two-sided<br>*Only common tests should be described solely by name; describe more complex techniques in the Methods section.*
☒	☐	A description of all covariates tested
☒	☐	A description of any assumptions or corrections, such as tests of normality and adjustment for multiple comparisons
☒	☐	A full description of the statistical parameters including central tendency (e.g. means) or other basic estimates (e.g. regression coefficient) AND variation (e.g. standard deviation) or associated estimates of uncertainty (e.g. confidence intervals)
☒	☐	For null hypothesis testing, the test statistic (e.g. *F*, *t*, *r*) with confidence intervals, effect sizes, degrees of freedom and *P* value noted<br>*Give P values as exact values whenever suitable.*
☒	☐	For Bayesian analysis, information on the choice of priors and Markov chain Monte Carlo settings
☒	☐	For hierarchical and complex designs, identification of the appropriate level for tests and full reporting of outcomes
☒	☐	Estimates of effect sizes (e.g. Cohen's *d*, Pearson's *r*), indicating how they were calculated

Our web collection on statistics for biologists contains articles on many of the points above.

Software and code

Policy information about availability of computer code

Data collection	Serial EM v3.6.11 was used for data collection.
Data analysis	MotionCor2 v1.4.0, CTFFIND4 v4.1.13, Relion3.1, Pymol v2.0.6, UCSF Chimera 1.11.2, ResMap v1.1.4, COOT 0.9.5, Phenix 1.19.2, Phenix.molprobity 1.19.2

For manuscripts utilizing custom algorithms or software that are central to the research but not yet described in published literature, software must be made available to editors and reviewers. We strongly encourage code deposition in a community repository (e.g. GitHub). See the Nature Research guidelines for submitting code & software for further information.

Data

Policy information about availability of data

All manuscripts must include a data availability statement. This statement should provide the following information, where applicable:

- Accession codes, unique identifiers, or web links for publicly available datasets
- A list of figures that have associated raw data
- A description of any restrictions on data availability

Cryo-EM density maps of DH-DDK relevant complexes have been deposited in the Electron Microscopy Data Bank (accession no. EMD-31684, EMD-31701, EMD-31685, EMD-31696, EMD-31686, EMD-31697,EMD-31688, EMD-31689, EMD-31690, EMD-31691, EMD-31692, EMD-31694, EMD-31695, EMD-31699, EMD-31670 and EMD-32355). Atomic coordinates have been deposited in the Protein Data Bank (ID codes 7V3U, 7V3V and 7W8G).

Field-specific reporting

Please select the one below that is the best fit for your research. If you are not sure, read the appropriate sections before making your selection.

☒ Life sciences ☐ Behavioural & social sciences ☐ Ecological, evolutionary & environmental sciences

For a reference copy of the document with all sections, see nature.com/documents/nr-reporting-summary-flat.pdf

Life sciences study design

All studies must disclose on these points even when the disclosure is negative.

Sample size	No statistical methods were used to predetermine sample size. All biochemical experiments were replicated two or more times. For structural data, the number of micrographs collected was determined based on the target resolution of the relevant electron microscopy 3D structures.
Data exclusions	Regarding the cryo-EM raw micrograph screening, exclusion was done based on the quality of the images. Regarding the particle selection, 2D and 3D classification were used and criterion is based on the quality of resulting 2D class average and 3D maps.
Replication	All attempts of replication were successful. Cryo-EM single particle analysis relies on averaging over a large number of independent observations.
Randomization	Samples were not allocated to groups.
Blinding	Investigators were not blinded during data acquisition and analysis because because visual inspection is necessary for the methods employed.

Reporting for specific materials, systems and methods

We require information from authors about some types of materials, experimental systems and methods used in many studies. Here, indicate whether each material, system or method listed is relevant to your study. If you are not sure if a list item applies to your research, read the appropriate section before selecting a response.

Materials & experimental systems

n/a	Involved in the study	
☐	☒	Antibodies
☐	☒	Eukaryotic cell lines
☒	☐	Palaeontology and archaeology
☒	☐	Animals and other organisms
☒	☐	Human research participants
☒	☐	Clinical data
☒	☐	Dual use research of concern

Methods

n/a	Involved in the study	
☒	☐	ChIP-seq
☒	☐	Flow cytometry
☒	☐	MRI-based neuroimaging

Antibodies

Antibodies used	MCM4 (Santa cruz, sc-166036, 1:1000 dilution), MCM6 (gift from Karim Labib)
Validation	Anti-Mcm4 Antibody (sc-166036), commercially available in Santa Cruz, is a mouse monoclonal IgG2a κ Mcm4 antibody, raised against amino acids 1-300 mapping at the N-terminus of Mcm4 of Saccharomyces cerevisiae origin. It is recommended for detection of Mcm4 of yeast origin by WB, IP, IF and ELISA by the manufacturer's website. This antibody has been validated by the vendor. Anti-MCM6 antibody is a gift from Karim Labib (School of Life Sciences, University of Dundee). Western blot was also done to validate the specificity of antibodies towards Mcm4 and Mcm6.

Eukaryotic cell lines

Policy information about cell lines

Cell line source(s)	Yeast W303-1a
Authentication	The cells were used only for protein purification, and not further authenticated.
Mycoplasma contamination	The cell line for purification was not tested.
Commonly misidentified lines (See ICLAC register)	No cell line used in this study was commonly misidentified lines.
